# Human umbilical cord mesenchymal stromal cell-derived exosomes from MSCs pretreated with inflammatory factors attenuate renal injury of diabetic mice by regulating macrophage polarization

**DOI:** 10.1042/CS20258827

**Published:** 2026-06-10

**Authors:** Chenhao Li, Yuan Tian, Ye Yuan, Yujun Du, Ziran Xu, Lisha Li

**Affiliations:** 1 Department of Nephrology, Lequn Branch, The First Hospital of Jilin University, Changchun, Jilin, China; 2 The Key Laboratory of Pathobiology, Ministry of Education, College of Basic Medical Sciences, Jilin University, Changchun, Jilin, China; 3 Department of Gynecology and Obstetrics, The Second Hospital of Jilin University, Changchun, Jilin, China; 4 Department of Clinical Laboratory, Lequn Branch, The First Hospital of Jilin University, Changchun, Jilin, China

**Keywords:** Diabetic nephropathy, Exosome, inflammation, macrophage polarization, Mesenchymal stromal cell, pretreat

## Abstract

Diabetic nephropathy (DN), a major complication of diabetes mellitus (DM), is characterized by severe clinical manifestations, impaired quality of life, and a high risk of progression to end-stage renal disease, underscoring the urgent need for effective therapeutic interventions. Mesenchymal stromal cell-derived exosomes (MSC-Exo) have emerged as promising candidates for mitigating inflammatory injury in DN due to their immunomodulatory properties, and exosomes derived from MSCs pretreated with inflammatory factors such as TNF-α and IFN-γ may possess enhanced therapeutic potential. In this study, exosomes isolated from human umbilical cord MSCs were characterized by transmission electron microscopy, nanoparticle tracking analysis, and western blotting. Their therapeutic effects were evaluated in diabetic mice, focusing on renal inflammation and macrophage polarization. Both normal MSC-Exo (Norm-Exo) and TNF-α&IFN-γ-pretreated MSC-Exo (TNF-α&IFN-γ-Exo) effectively ameliorated kidney injury and promoted M2 macrophage polarization, with TNF-α&IFN-γ-Exo showing superior efficacy. High-glucose-stimulated RAW264.7 cells were used to explore the underlying mechanisms, and high-throughput RNA sequencing identified inhibitor of DNA binding 3 (ID3) as a molecule involved in MSC-Exo-regulated macrophage polarization. Loss-of-function experiments confirmed that ID3 knockdown alone recapitulated the effects of exosomes, promoting M2 polarization and suppressing M1 markers. Conversely, ID3 overexpression attenuated exosome efficacy. Mechanistically, ID3 partially mediated exosome-induced inhibition of the NF-κB pathway. The translational relevance of these findings was further validated in PMA-differentiated THP-1 human macrophages. Collectively, these findings demonstrate that MSC-Exo—particularly TNF-α&IFN-γ-Exo—attenuate diabetic renal injury by modulating macrophage polarization through ID3 regulation, highlighting a novel cell-free immunomodulatory approach for DN therapy.

## Introduction

Diabetic nephropathy (DN) is one of the most severe microvascular complications of diabetes mellitus (DM), often progressing to end-stage renal disease (ESRD) [1–3]. Patients with DN face a diminished quality of life, substantial economic burdens, and often require lifelong renal replacement therapy, underscoring the urgent need to elucidate its pathogenesis and develop effective treatments.

DM encompasses type 1 diabetes (T1DM), type 2 diabetes (T2DM), and rare forms, with T1DM and T2DM being the most prevalent [4]. Both types are associated with systemic inflammation, which contributes to the development of DN [5]. Notably, approximately 30% of T1DM patients and 40% of T2DM patients progress to DN [6–9]. In DN pathogenesis, systemic and intrarenal inflammation play pivotal roles, with the severity of renal injury closely linked to the intensity of inflammatory responses [10,11].

Macrophages, as key innate immune cells, are critically involved in DN progression. The extent of renal macrophage infiltration strongly correlates with disease severity [12]. Macrophages exhibit remarkable plasticity, polarizing into two primary phenotypes: proinflammatory type 1 (M1) and anti-inflammatory type 2 (M2) macrophages [13]. In DN, M1 macrophages exacerbate inflammation, whereas M2 macrophages mitigate immune-mediated damage [14–16]. Thus, therapeutic strategies targeting macrophage polarization to modulate inflammation hold significant promise for DN treatment.

Mesenchymal stromal cells (MSCs) have emerged as potential therapeutics for inflammatory diseases due to their dual capacities for tissue repair and immunomodulation [17,18]. While exogenous MSCs can engraft at injury sites to promote regeneration, their paracrine effects—particularly via secreted exosomes (MSC-Exo)—are increasingly recognized as central to their therapeutic benefits [19]. Compared to whole MSCs, MSC-Exo offer advantages such as biochemical stability, reduced toxicity, and easier storage [17,20]. These vesicles modulate immune responses by suppressing proinflammatory cells (e.g. monocytes, M1 macrophages, Th1/Th17 cells) while promoting anti-inflammatory populations (e.g. M2 macrophages, Treg cells) [21–23]. Importantly, pretreatment of MSCs with inflammatory cytokines like TNF-α and IFN-γ enhances the immunomodulatory potency of their exosomes (TNF-α&IFN-γ-Exo) [24–27], positioning MSC-Exo as a promising intervention for DN-associated inflammation.

Although the role of mesenchymal stem cell–derived exosomes (MSC-Exo) in regulating macrophage polarization in DN has been increasingly recognized [28], previous studies have predominantly focused on exosomal miRNAs, inflammatory mediators, and downstream signaling pathways such as NF-κB and STAT3 [29–32]. However, upstream transcriptional regulators that directly govern macrophage polarization in this context remain largely unexplored. In the present study, we identify ID3 as a previously unrecognized regulatory factor under high-glucose (HG) conditions and demonstrate its involvement in MSC-Exo-mediated macrophage polarization. These findings extend the current mechanistic framework by introducing a transcriptional regulatory layer that complements existing knowledge of exosome-mediated immunomodulation.

However, the mechanisms by which MSC-Exo regulates DN inflammation—particularly through macrophage polarization—remain unclear, and whether cytokine pretreatment augments this effect is unknown. Addressing these questions could reveal novel therapeutic targets for DN. In this study, we isolated and characterized exosomes from human umbilical cord MSCs (HUCMSCs) and demonstrated that both normal MSC-Exo (Norm-Exo) and TNF-α&IFN-γ-Exo ameliorated hyperglycemia, reduced urinary albumin-to-creatinine ratios (UACR), and attenuated renal inflammation in DM mice, with TNF-α&IFN-γ-Exo showing superior efficacy. Through RNA sequencing and functional validation, we identified inhibitor of DNA binding 3 (ID3) as a mediator involved in MSC-Exo-regulated macrophage polarization under HG conditions. Using both gain-of-function (overexpression) and loss-of-function (siRNA-mediated knockdown) approaches, we demonstrated that ID3 is necessary for the full immunomodulatory effect of exosomes. Mechanistically, ID3 partially mediated exosome-induced suppression of the NF-κB pathway. The translational relevance of these findings was further validated in human THP-1 macrophages. These results provide a mechanistic foundation for future DN therapies.

## Materials and methods

### Isolation and identification of HUCMSC-derived exosomes

HUCMSCs (HyCyte™) were cultured in DMEM-F12 medium. When cells reached 70%–80% confluence, the culture medium was replaced with DMEM-F12 containing 5% exosome-depleted FBS (Sigma), supplemented with IFN-γ (10 ng/ml) and TNF-α (10 ng/ml) (Sino Biological Inc), and cultured for an additional 48 h.

Exosomes were isolated from the supernatant using differential ultracentrifugation. Briefly, the supernatant was centrifuged at 10,000 × g for 45 min, followed by ultracentrifugation at 120,000 × g for 2 h. The resulting pellet was resuspended in sterile PBS. Exosome ultrastructure and morphology were confirmed by transmission electron microscopy (TEM; FEI Tecnai 12). Nanoparticle tracking analysis (NTA; ZetaView PMX 120) was used to determine size distribution and concentration. Western blot (WB) analysis verified the presence of exosomal surface markers CD9 (ABclonal), CD63 (ABclonal), and TSG101 (Abcam).

### Establishment of T1DM model and treatment

All experimental procedures were conducted in accordance with relevant guidelines and regulations, following a protocol approved by the Committee of Experimental Animal Ethics of Jilin University, Changchun, China (Approval number: 2022-180; Date: 1 March 2022) and in compliance with ARRIVE guidelines 2.0 (https://www.arriveguidelines.org). Four groups of male BALB/c mice (6–8 weeks old) were housed under controlled conditions (12-h light/dark cycle, 22°C–25°C, 50%–60% humidity) at the Animal Center of the School of Basic Medical Sciences, Jilin University, China. T1DM was induced by intraperitoneal injection of streptozotocin (STZ; 60 mg/kg) for 5 consecutive days. Diabetes was confirmed by measuring blood glucose levels ≥16.7 mmol/l on three consecutive occasions one week post-induction.

The experimental groups included: negative control group (NC group), diabetes mellitus group (DM group), diabetes mellitus + Norm-Exo intervention group (DM + Norm-Exo group), diabetes mellitus + pretreated MSC-Exo intervention group (DM + TNF-α&IFN-γ-Exo group). At 12 weeks post-T1DM induction, mice received tail vein injections of either Norm-Exo (50 μg/mouse) or TNF-α&IFN-γ-Exo (50 μg/mouse) twice weekly for 5 weeks. Control groups received equivalent volumes of PBS. Eighteen weeks after diabetes confirmation, all animals were killed by cervical dislocation for tissue collection. Spleen and kidney samples were harvested for WB, histological analysis, and flow cytometry.

### Details of animal euthanasia and anesthesia

No anesthetic agents were administered during experimental procedures. Euthanasia was performed exclusively by cervical dislocation, without the use of chemical agents.

### Histopathology analysis and immunohistochemical staining

Kidney tissues were sectioned longitudinally, fixed in 10% neutral buffered formalin, paraffin-embedded, and cut into 2-μm sections. Histopathological evaluation was performed using hematoxylin and eosin (H&E), periodic acid-Schiff (PAS), and Masson’s trichrome (MTS) staining to assess tissue morphology and fibrosis.

For immunohistochemical staining, paraffin sections were deparaffinized in xylene and rehydrated through graded alcohol solutions. Antigen retrieval was performed using sodium citrate buffer under pressure, with endogenous peroxidase and non-specific binding blocked using reagents from MXB Biotechnologies. Sections were incubated overnight at 4°C with primary antibodies against TNF-α, IL-10 (1:200; ABclonal), IL-1β, and IL-6 (1:100; ABclonal). Following PBS washes, sections were incubated with goat anti-rabbit IgG secondary antibody (MXB Biotechnologies), developed with 3,3‘-Diaminobenzidine (DAB), and counterstained with hematoxylin. After dehydration through graded ethanol and xylene, sections were mounted with neutral resin for microscopic examination. All primary antibodies are listed in Supplementary Table S1.

### High‐glucose stimulation and HUCMSC-derived exosomes treatment in RAW 264.7 cells

RAW 264.7 cells were thawed and maintained in RPMI 1640 medium (Gibco) at 37°C in a humidified 5% CO_2_ incubator. The experimental design comprised four treatment groups: (1) NC group (PBS-treated control), (2) HG group (35 mM glucose for 48 h), (3) HG + Norm-Exo group (35 mM glucose followed by 10 μg/ml Norm-Exo for 48 h), and (4) HG + TNF-α&IFN-γ-Exo group (35 mM glucose followed by 10 μg/ml TNF-α&IFN-γ-Exo for 48 h).

Cells were cultured under normal glucose (5.5 mM) or high glucose (35 mM) conditions. For osmotic control experiments, an iso-osmotic control medium was prepared by supplementing normal glucose medium with 23.9 mM mannitol to match the osmolarity of the HG condition.

### Sample preparation and unique identifier RNA-seq methods

High-throughput unique identifier (UID) RNA sequencing was performed on three experimental groups (HG, HG + Norm-Exo, and HG + TNF-α&IFN-γ-Exo), each containing three biological replicates. For sample preparation, cells were detached using a cell scraper, suspended in PBS, and centrifuged at 500 × g for 10 min at 4 °C. After supernatant removal, cell pellets were flash-frozen in liquid nitrogen and stored at −80 °C.

RNA extraction was performed using TRIzol reagent, with quality assessed by Nanodrop spectrophotometry (Thermo Fisher Scientific) and agarose gel electrophoresis. RNA quantification was conducted using Qubit 3.0 with the Qubit RNA Broad Range Assay kit (Life Technologies). Stranded RNA sequencing libraries were prepared from 2 μg total RNA using the KC-Digital™ Stranded mRNA Library Prep Kit (Wuhan Seqhealth), incorporating 8-base UMI tags to eliminate PCR duplication bias. Size-selected libraries (200-500 bp) were sequenced on the DNBSEQ-T7 platform (MGI Tech) using 150 bp paired-end reads.

Bioinformatic analysis included: (1) quality control using Trimmomatic v0.36, (2) UMI-based error correction, (3) alignment to the reference genome using STAR v2.5.3a, (4) gene expression quantification with featureCounts v1.5.1, and (5) differential expression analysis using edgeR v3.12.1. Functional annotation was performed with KOBAS v2.1.1 for gene ontology (GO) and kyoto encyclopedia of genes and genomes (KEGG) pathway analysis. Complete RNA-seq datasets are provided in Supplementary File ‘Supplementary file_RNA sequencing data_ All_Samples_rpkm.xls’. Differentially expressed genes (DEGs) were identified based on an false discovery rate (FDR) threshold of <0.05 after Benjamini–Hochberg correction.

### WB

Protein extracts from kidney tissues and RAW 264.7 cells were prepared using RIPA buffer supplemented with PMSF (Beyotime) or a Nucleoprotein Extraction Kit (Sangon Biotech). Protein concentrations were determined by BCA assay (Beyotime). Samples (20–50 μg) were separated on 8%–15% SDS–PAGE gels (Yeasen Biotech) and transferred to PVDF membranes. After blocking with 5% non-fat milk, membranes were incubated overnight at 4°C with primary antibodies (1:1000 dilution) against TSG101 (Abcam), CD9, CD63, TNF-α, IL-1β, IL-10, IL-6 (ABclonal), β-actin (Servicebio), ID3, Arg1 (Santa Cruz), iNOS (Cell Signaling Technology), and Lamin A (Boster). Following incubation with HRP-conjugated secondary antibodies (1:2000), protein bands were visualized using an ECL kit (MedChemExpress) and quantified with ImageJ software.

### Quantitative real-time PCR

Total RNA was extracted using the Ultrapure RNA Kit (Cwbio) and reverse transcribed with HiFiScript gDNA Removal RT MasterMix (Cwbio). qPCR was performed on a 7300 Plus Real-Time PCR System (Applied Biosystems) using MagicSYBR Mixture (Cwbio). Relative mRNA expression was calculated using the 2^(−ΔΔCt)^ method with β-actin as the endogenous control. The primer sequences used are listed in Supplementary Table S2.

### Flow cytometry

Spleen tissues were mechanically dissociated and filtered through a 200-μm mesh. After RBC lysis (Beyotime), cells were stained with fluorescent antibodies against CD3-FITC, CD4-APC (Sungene Biotech), CD25-FITC, lineage cocktail (Ly6G/CD3/B220-APC), CD115-PE/Cy5.5 (eBioscience), and CD11b-PE (BioLegend). Intracellular staining for IFN-γ-PE/Cy7, IL-4-PE, IL-17-FITC, and FoxP3-PE (BioLegend) was performed following fixation/permeabilization (BioLegend reagents).

Kidney tissues were digested with 0.2% collagenase IV (Beyotime) and processed similarly. Macrophage populations were identified using F4/80-APC, CD11b-PE, CD86-FITC, and intracellular CD206-PE/Cy7 (BioLegend). Data were acquired on a BD flow cytometer and analyzed using FlowJo software.

### Transfection

RAW 264.7 cells in 6-well plates were transfected with either empty vector or ID3 expression plasmid (GeneChem) using Advanced DNA RNA Transfection Reagent (ZetaLife). After 24 h, cells received one of three treatments: (1) high glucose (35 mM, 48 h), (2) exosomes (10 μg/ml Norm-Exo or TNF-α&IFN-γ-Exo, 48 h), or (3) sequential high glucose (48 h) followed by exosome treatment (48 h).

For gene silencing experiments, RAW264.7 and THP-1 cells were transfected with siRNA (final concentration 30 nM) using RNATransMate reagent (BBI) according to the manufacturer’s instructions. Cells were cultured for 16 h before subsequent treatments or analyses. Three siRNA duplexes targeting human ID3 and three targeting mouse Id3 were designed (Supplementary Table S3), and the most efficient sequence was selected based on quantitative real-time PCR (qRT-PCR).

For THP-1 differentiation, cells were treated with 100 ng/ml Phorbol 12-Myristate 13-Acetate (PMA) for 24 h to induce adherence, followed by recovery in fresh medium prior to transfection. Polarization experiments were performed under normal glucose (NG, 5.5 mM) or high glucose (HG, 35 mM) conditions, and gene expression was analyzed by qRT-PCR.

### Statistical analysis

All quantitative data are presented as mean ± SEM. The sample size (*n*) for each experiment is indicated in the corresponding figure legends. For *in vivo* experiments, *n* = 5 mice per group. For *in vitro* experiments, at least three independent biological replicates (*n* = 3) were performed.

Statistical analyses were conducted using GraphPad Prism 8.3.0 and SPSS 29.0. Comparisons between two groups were performed using unpaired *t*-test. For multiple group comparisons, one-way ANOVA with Tukey’s post-hoc test.

For RNA-seq data, differential expression analysis was performed using the edgeR package. *P*-values were adjusted for multiple testing using the Benjamini–Hochberg method to control the FDR. Genes with FDR <0.05 were considered statistically significant. *P*-value <0.05 was considered statistically significant.

## Results

### Characterization of HUCMSC-Exo

**Figure 1:**
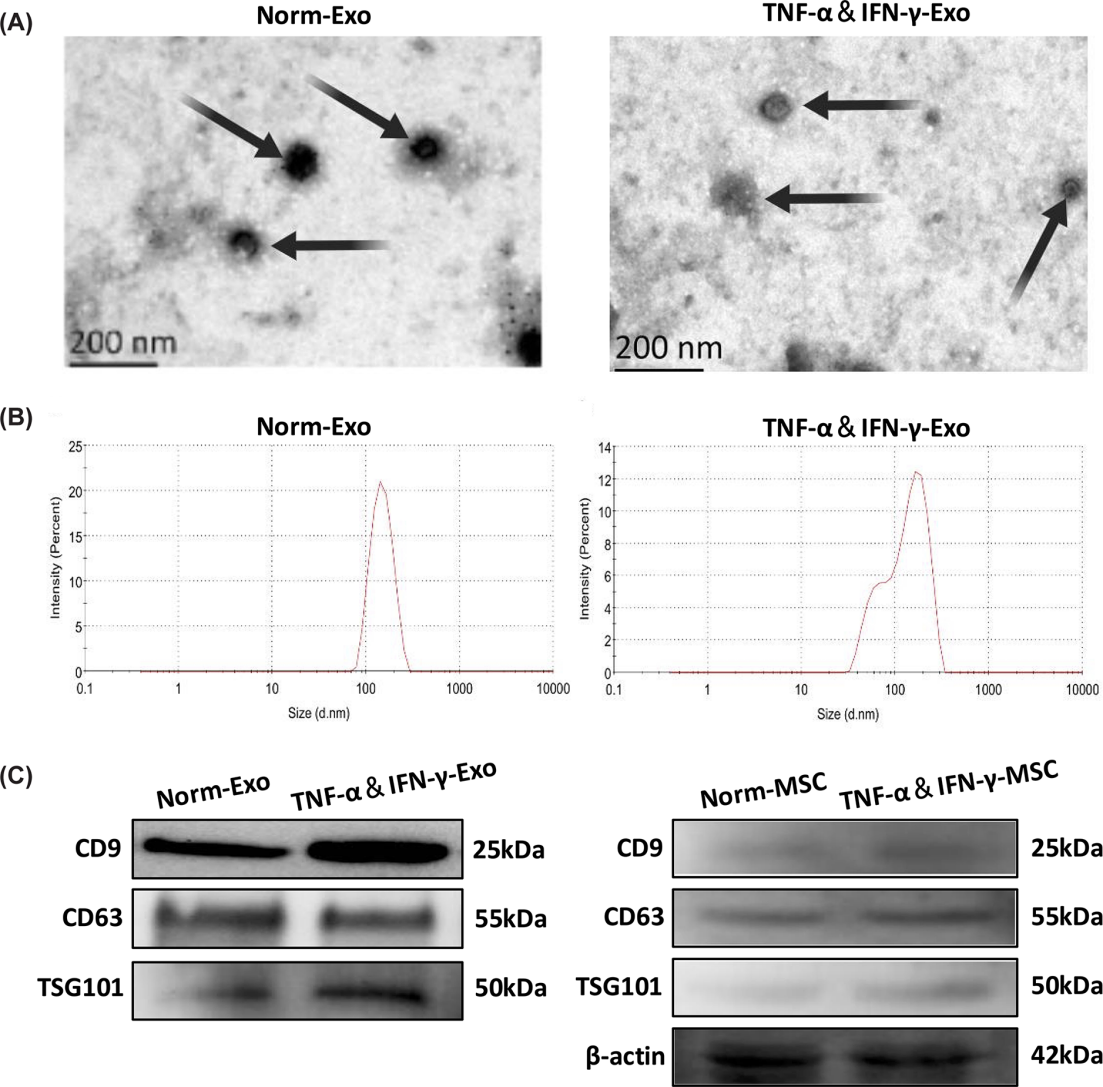
Characterization of HUCMSC-Exo (**A**) TEM analysis of Norm-Exo and TNF-α&IFN-γ-Exo (scale bar = 200 nm). (**B**) NTA of Norm-Exo and TNF-α&IFN-γ-Exo. (**C**) WB confirmed that Norm-Exo and TNF-α&IFN-γ-Exo express marker proteins CD9, CD63, and TSG101 (*n* = 3), full-length uncropped blots are shown in Supplementary Figure S7.

Two types of HUCMSC-derived exosomes (Norm-Exo and TNF-α&IFN-γ-Exo) were successfully isolated via ultracentrifugation and characterized using TEM, NTA, and WB. TEM analysis confirmed that both Norm-Exo and TNF-α&IFN-γ-Exo exhibited a typical spherical morphology with double membranes (Figure 1A). NTA revealed peak diameters of 184.4 nm for Norm-Exo and 173 nm for TNF-α&IFN-γ-Exo (Figure 1B). WB further confirmed the presence of exosomal markers CD9, CD63, and TSG101 in both exosome types (Figure 1C). These findings demonstrate the successful isolation and characterization of HUCMSC-derived exosomes.

### The effects of HUCMSC-Exo on conventional indicators of T1DM mice

**Figure 2:**
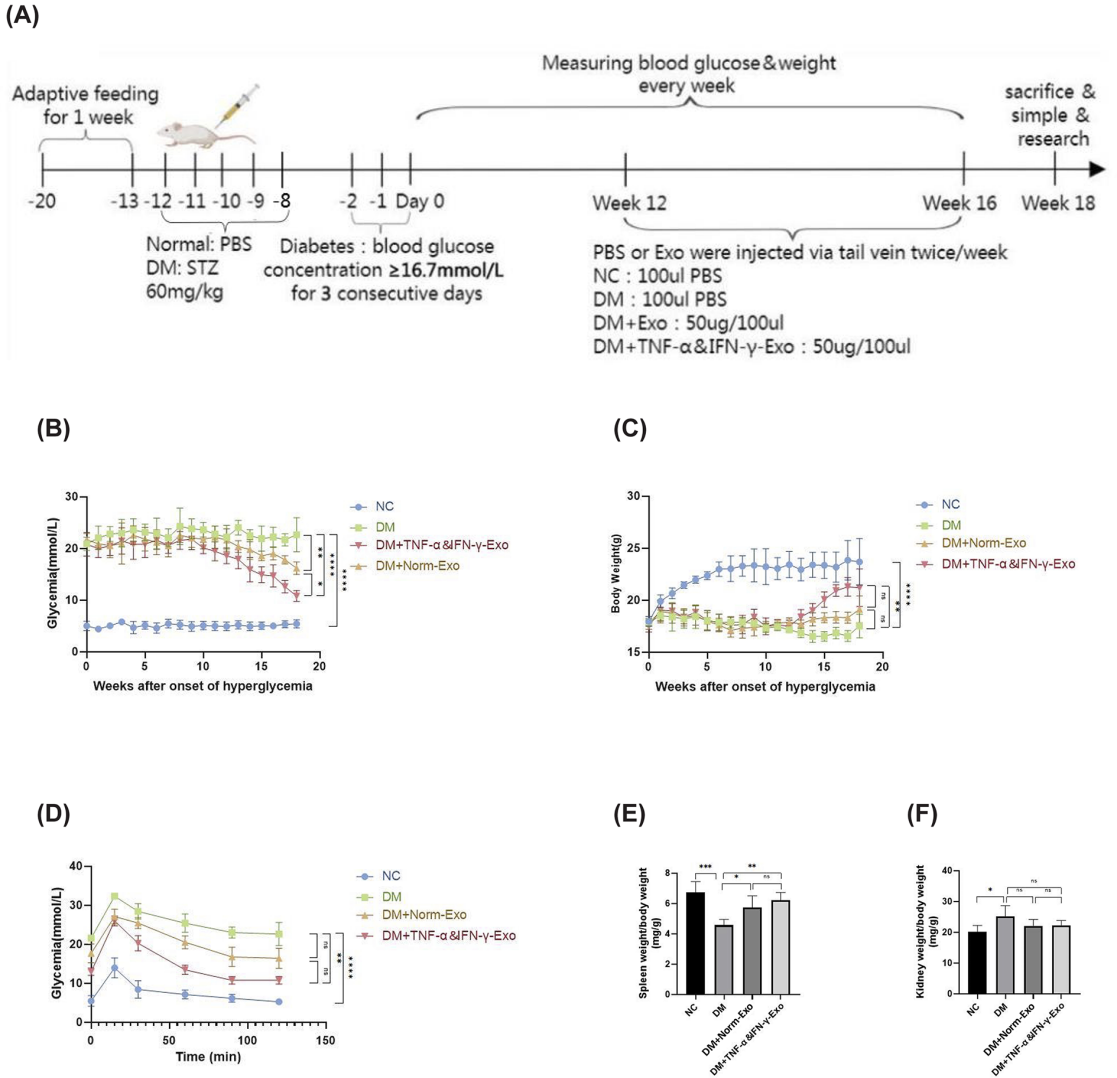
Flow diagram of experiments and conventional indicators of T1DM mice (**A**) Timetable and flowchart of animal experiment. (**B–F**) The change of conventional indicators of T1DM mice (blood glucose levels, body weight, glucose tolerance, spleen coefficient, and kidney coefficient) in response to Norm-Exo and TNF-α&IFN-γ-Exo; (*n* = 5), Statistical comparisons were performed using one-way ANOVA followed by Tukey’s post hoc test, ns indicates no significant difference *P* > 0.05, *P < 0.05, **P < 0.01, ***P < 0.001, ****P < 0.0001.

STZ-induced T1DM mice were treated with exosomes via tail vein injection (Figure 2A). Both Norm-Exo and TNF-α&IFN-γ-Exo significantly reduced fasting blood glucose levels, with TNF-α&IFN-γ-Exo exhibiting a more pronounced effect (Figure 2B). While TNF-α&IFN-γ-Exo significantly increased body weight in diabetic mice (*P* < 0.05), Norm-Exo did not show a statistically significant effect (*P* > 0.05) (Figure 2C). Both exosome treatments improved glucose tolerance, though no significant difference was observed between the two (*P* > 0.05) (Figure 2D). Additionally, both exosomes attenuated the decrease in spleen coefficient (Figure 2E) but had no significant impact on kidney coefficient (Figure 2F).

### Effects of HUCMSC-Exo on CD4^+^ T cells and its subtypes in T1DM mice

**Figure 3:**
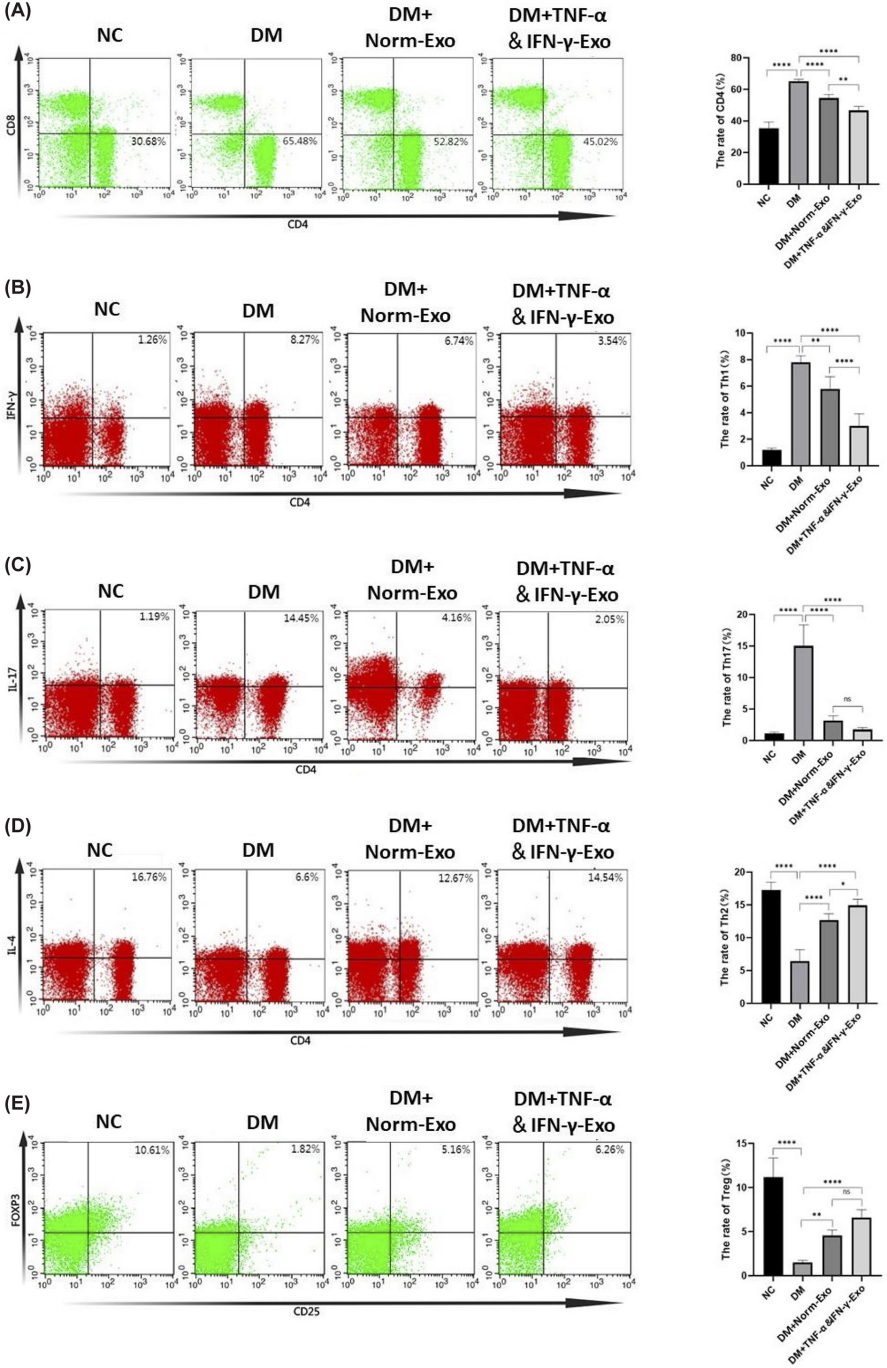
Effects of HUCMSC-Exo on CD4^+^ T cells and its subtypes in T1DM mice (**A**) Changes in the proportion of CD4^+^ T cells (CD4^+^) in the spleen was determined by Flow cytometry. (**B-E**) Changes in the proportion of CD4^+^ T cells’ subtypes in the spleen was determined by Flow cytometry. Th1 cells: CD4^+^ and IFN-γ^+^ (**B**); Th17 cells: CD4^+^ and IL-17^+^ (**C**); Th2 cells: CD4^+^ and IL-4^+^ (**D**); Treg cells: CD4^+^, CD25^+^ and Foxp3^+^. (**E**) (*n* = 5), statistical analysis was conducted using one-way ANOVA with Tukey’s post hoc test for multiple comparisons, ns indicates no significant difference, *P* > 0.05, *P < 0.05, **P < 0.01, ****P < 0.0001. Data are presented relative to the non-diabetic control (NC) group, which serves as the physiological baseline.

The immunological mechanism of T1DM involves CD4^+^ T cells and their subtypes, The changes of them may serve as indicators of the inflammatory response dynamics in the progression of T1DM. Flow cytometry was employed to assess changes in CD4^+^ T cells and their subsets (Th1, Th2, Th17, and Tregs) in the spleens of experimental mice (Supplementary Figure S1). Compared with the non-diabetic control (NC) group, diabetic mice exhibited a significant increase in the proportion of CD4^+^ T cells (Figure 3A) and a shift toward a pro-inflammatory phenotype, characterized by elevated Th1 and Th17 populations and reduced Th2 and Treg cells. Treatment with both Norm-Exo and TNF-α&IFN-γ-Exo partially reversed these alterations, reducing Th1 and Th17 cells (Figure 3B,C) while increasing Th2 and Treg populations (Figure 3D,E), thereby restoring immune balance toward non-diabetic baseline levels. Notably, TNF-α&IFN-γ-Exo demonstrated a more pronounced regulatory effect.

### HUCMSC-Exo ameliorated inflammation of renal and regulated macrophage polarization in T1DM mice

**Figure 4:**
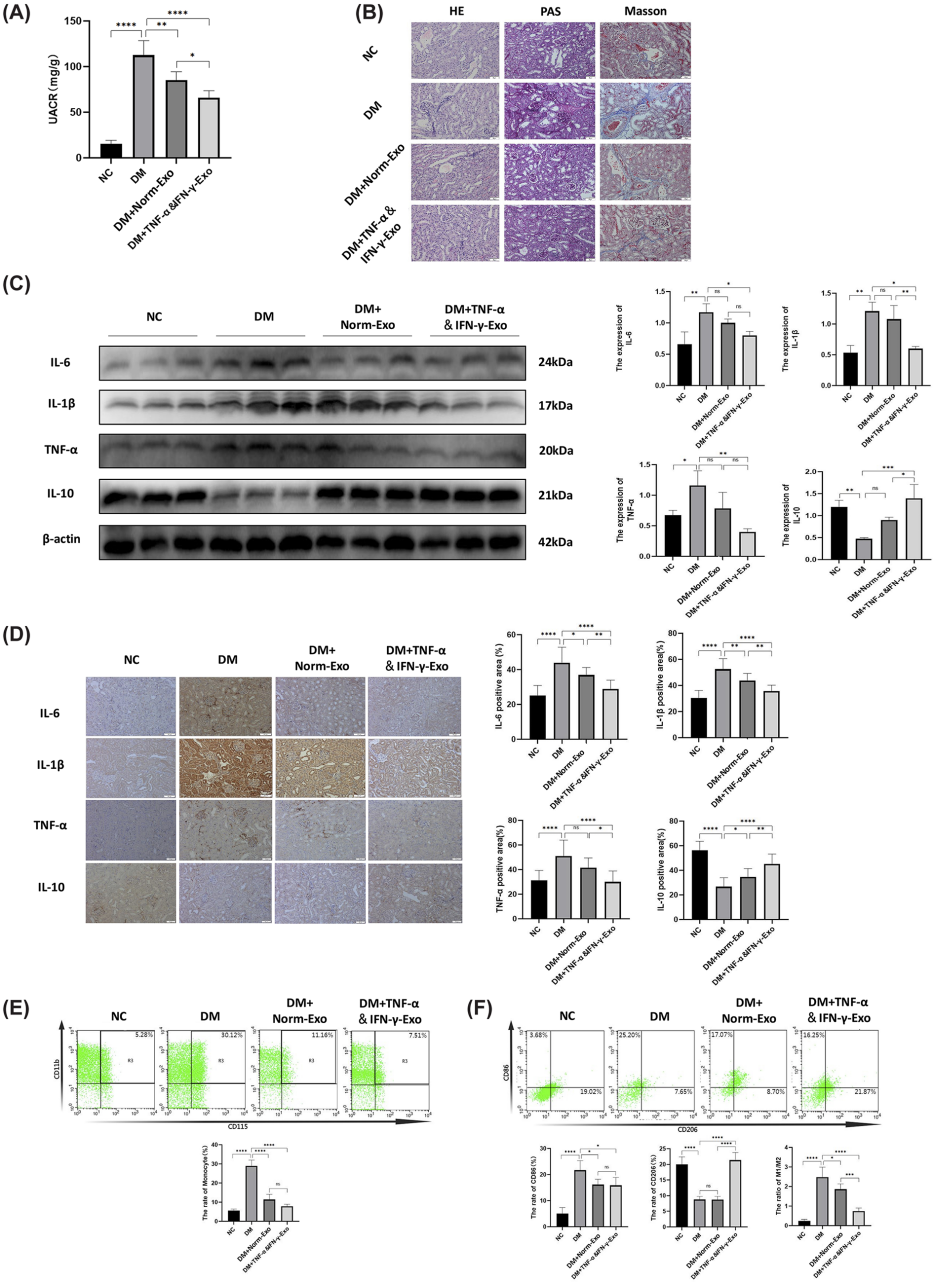
HUCMSC-Exo ameliorated inflammation of renal and regulated macrophage polarization in T1DM mice (**A**) The change of UACR. (**B**) HE, PAS, and MTS on renal tissue. Scale Bar = 50 μm. (**C**) WB and quantification results of IL-6, IL-1β, TNF-α, and IL-10 in kidney tissue of mice. (**D**) The expression levels of pro-inflammatory cytokines IL-6, IL-1β, TNF-α, and anti-inflammatory cytokines IL-10 in renal tissues were detected by Immunohistochemical staining and their positive area was evaluated by ImageJ. Scale Bar = 50 μm. (**E**) Changes in the proportion of monocytes (Ly6G^−^, CD3^−^, B220^−^, and CD11b^+^, CD115^+^) in the spleen was determined by Flow cytometry. (**F**) Changes in the proportion of macrophage (CD11b^+^ and F4/80^+^) and its subtypes (M1(CD86^+^) and M2(CD206^+^)) in the renal was determined by Flow cytometry. (*n* = 5), statistical differences among groups were analyzed using one-way ANOVA followed by Tukey’s post hoc test, ns indicates no significant difference, *P* > 0.05, *P < 0.05, **P < 0.01, ***P < 0.001, ****P < 0.0001. Full-length uncropped blots are shown in Supplementary Figure S8. Data are presented relative to the non-diabetic control (NC) group, which serves as the physiological baseline.

To evaluate renal protection, UACR and histopathological analyses (H&E, PAS, MTS) were performed. Both exosome types reduced UACR, with TNF-α&IFN-γ-Exo exhibiting greater efficacy (Figure 4A). Histologically, diabetic kidneys showed inflammatory cell infiltration, glomerular basement membrane thickening, and collagen deposition, all of which were ameliorated by exosome treatment (Figure 4B).

Compared with the NC group, diabetic mice exhibited elevated levels of pro-inflammatory cytokines (IL-6, IL-1β, TNF-α) and reduced anti-inflammatory IL-10 expression in renal tissues. Treatment with both Norm-Exo and TNF-α&IFN-γ-Exo significantly attenuated these changes, decreasing pro-inflammatory cytokines and restoring IL-10 levels toward those observed in non-diabetic controls (Figure 4C,D). Flow cytometry analysis showed that, compared with the NC group, diabetic mice displayed increased splenic monocyte recruitment and a shift in renal macrophage polarization toward the pro-inflammatory M1 phenotype. Exosome treatment reduced monocyte proportions (Figure 4E) and promoted macrophage polarization toward the M2 phenotype (Figure 4F), thereby partially restoring these parameters toward non-diabetic baseline levels. Notably, TNF-α&IFN-γ-Exo exerted a more pronounced effect on M2 polarization.

To further determine whether the renoprotective effects of exosome treatment are associated with glycaemic control, correlation analyses were performed between fasting blood glucose levels and renal injury as well as inflammatory markers. Fasting blood glucose levels showed a strong positive correlation with UACR (*r* = 0.8772, *R*^2^ = 0.7695, *P* < 0.0001) and IL-6 levels (*r* = 0.8090, *R*^2^ = 0.6545, *P* < 0.0001), and moderate positive correlations with IL-1β (*r* = 0.5354, *P* = 0.0150) and TNF-α (*r* = 0.5780, *P* = 0.0076). In contrast, fasting blood glucose was negatively correlated with the anti-inflammatory cytokine IL-10 (*r* = −0.6850, *R*^2^ = 0.4692, *P* = 0.0009) (Supplementary Figure S2). These results indicate that improved glycaemic control is closely associated with reduced renal injury and inflammation.

### MSC-Exo exhibited polarization regulatory effects on High‐glucose stimulated RAW 264.7 cells

**Figure 5:**
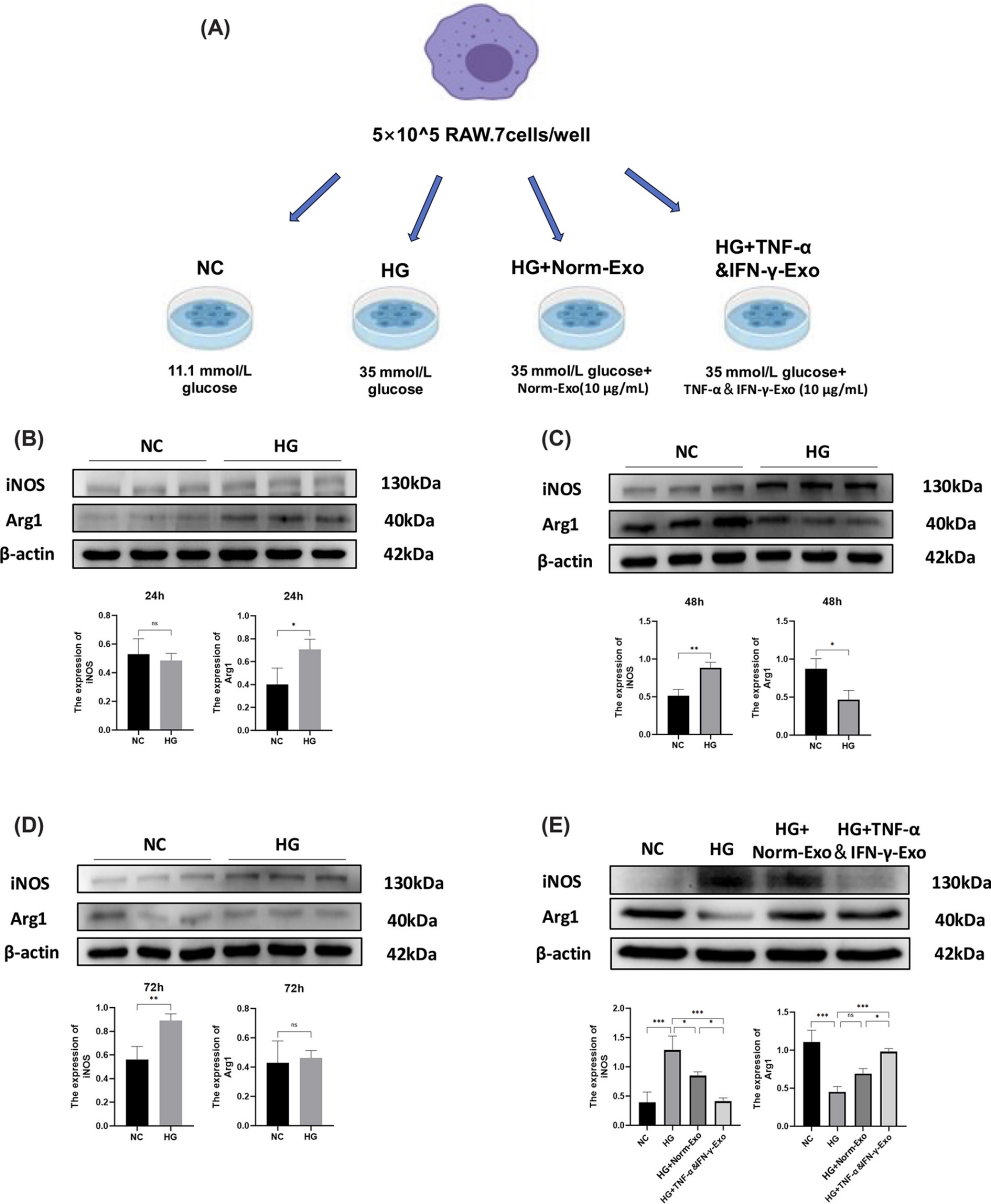
MSC-Exo exhibited polarization regulatory effects on high‐glucose stimulatedRAW264.7 cells (**A**) Grouping and processing methods of cell experiment. (**B–D**) WB and quantification results of iNOS and Arg1 in RAW264.7 cells cultured with high glucose (35 mmol/l) for 24 h, 48 h, and 72 h (full-length uncropped blots are shown in Supplementary Figure S9). (**E**) WB and quantification results of iNOS and Arg1 in RAW264.7 cells (full-length uncropped blots are shown in Supplementary Figure S10). (*n* = 3). Two groups were performed using unpaired *t*-test. For multiple group comparisons, one-way ANOVA with Tukey’s post-hoc test, ns indicates no significant difference, *P* > 0.05, *P < 0.05, **P < 0.01, ***P < 0.001.

*In vitro*, high glucose (35 mM) increased M1 marker (iNOS) and decreased M2 marker (Arg1) expression in RAW 264.7 cells, with maximal effects at 48 h (Figure 5B-D). We grouped and intervened RAW264.7 cells according to the method in Figure 5A. Both Norm-Exo and TNF-α&IFN-γ-Exo suppressed iNOS and enhanced Arg1 expression, with TNF-α&IFN-γ-Exo showing greater efficacy (Figure 5E), confirming their role in promoting M2 polarization.

To exclude nonspecific osmotic effects induced by high glucose, an iso-osmotic control using mannitol was included. As shown in Supplementary Figure S3, mannitol treatment did not reproduce the effects of high glucose on macrophage polarization markers. Specifically, iNOS expression remained comparable to the normal glucose group, while Arg1 levels were not significantly reduced. These results indicate that the observed alterations in macrophage polarization are attributable to high glucose rather than osmotic stress.

### UID RNA-seq reveals changes of HUCMSC-Exo on the mRNA expression profile in high‐glucose-stimulated RAW264.7 cells

RNA-seq analysis identified 291 DEGs in the HG + Norm-Exo group (174 upregulated, 117 downregulated) and 90 DEGs in the HG + TNF-α&IFN-γ-Exo group (41 upregulated, 49 downregulated) compared to the HG group. Among the 121 differential genes, 41 genes were upregulated, and 80 genes were downregulated in HG + TNF-α&IFN-γ-Exo group compared with HG + Norm-Exo group (Supplementary Figure S4A1,A3). ID3 expression was downregulated in the HG + Exo and HG + TNF-α&IFN-γ-Exo groups compared with the HG group (logFC = −0.67, *P* = 0.00095, FDR = 0.0145; logFC = −0.81, *P* = 0.00004, FDR = 0.0262), with differences remaining significant after multiple testing correction.

Cluster analysis confirmed distinct gene expression profiles among groups (Supplementary Figure S4B). GO and KEGG analyses highlighted significant alterations in transcriptional regulation and inflammation-related pathways (Supplementary Figure S4C,D). Among them, the items related to transcriptional regulation and cellular metabolism exhibited significant alterations. In the KEGG pathway analysis on signal transduction level, some oxidative stress or inflammation-associated signaling pathways down-regulated, such as NOD-like receptor signaling pathway and AGE-RAGE signaling pathway (Supplementary Figure S4E,F).

### ID3 was involved in MSC-Exo mediated macrophage polarization

**Figure 6:**
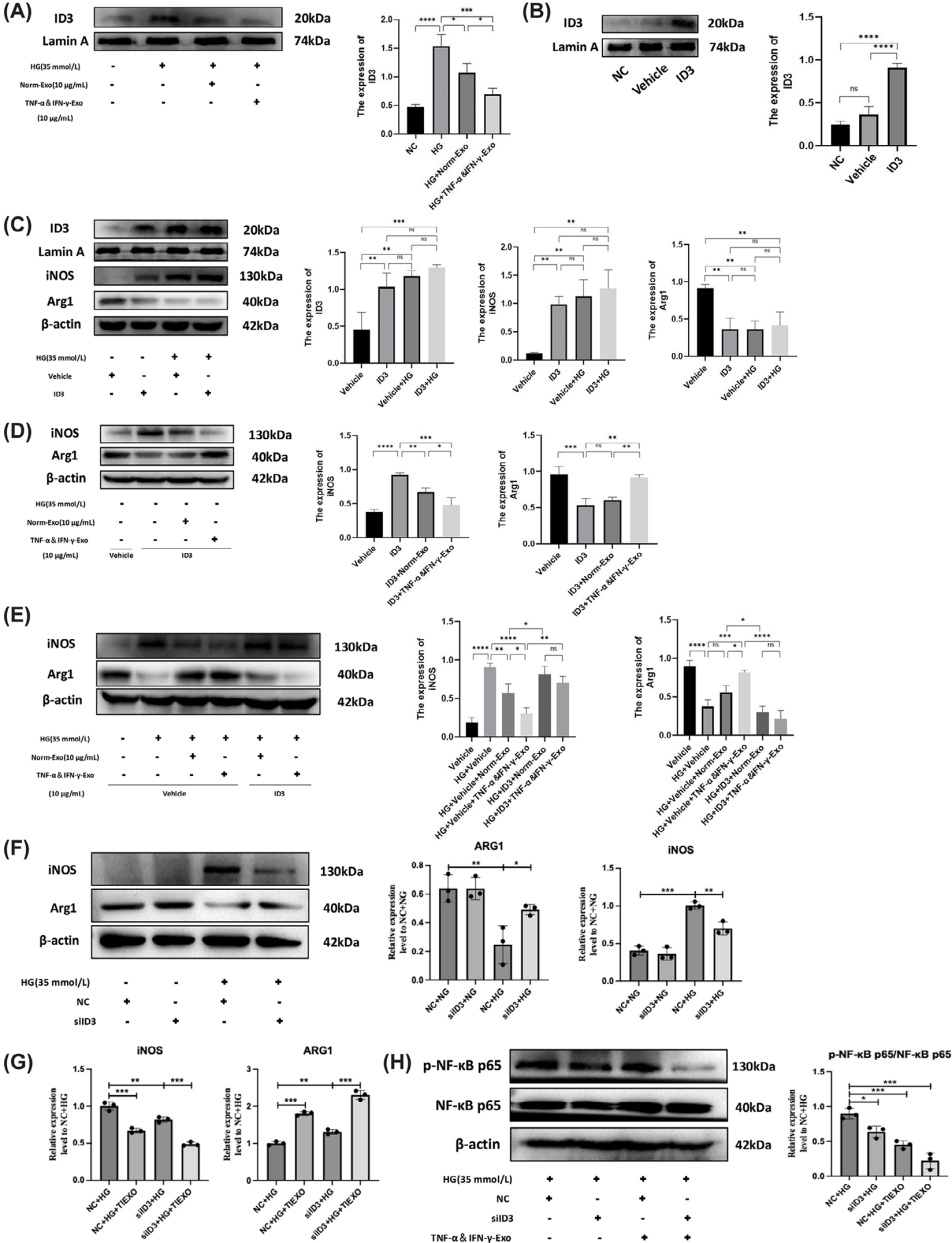
ID3 was involved in Exo-mediated macrophage polarization (**A**) WB confirmed the expression of ID3 was upregulated in high glucose (HG, 35 mM) group and was downregulated after Norm-Exo and TNF-α&IFN-γ-Exo treatment (full-length uncropped blots are shown in Supplementary Figure S11). (**B**) ID3 overexpression transfection of RAW 264.7 cells. The expression level of ID3 was detected using WB and quantified by ImageJ (full-length uncropped blots are shown in Supplementary Figure S12). (**C**) After ID3 overexpression transfection of RAW 264.7 cells for 24 h, followed by cultured with HG (35 mM) for 48 h. The expression level of ID3, iNOS, and Arg1 was detected using WB and quantified by ImageJ (full-length uncropped blots are shown in Supplementary Figure S13). (**D**) After ID3 overexpression transfection of RAW 264.7 cells for 24 h, followed by treatment with Norm-Exo and TNF-α&IFN-γ-Exo for 48 h. The expression level of iNOS and Arg1 was detected using WB and quantified by ImageJ (full-length uncropped blots are shown in Supplementary Figure S14). (**E**) After ID3 overexpression transfection of RAW 264.7 cells for 24 h, followed by cultured with HG (35 mM) for 48 h, then treated with Norm-Exo and TNF-α&IFN-γ-Exo for 48 h. The expression level of iNOS and Arg1 was detected using WB and quantified by ImageJ (full-length uncropped blots are shown in Supplementary Figure S15). (**F**) Cells were transfected with si-NC or si-ID3 and treated with normal glucose (NG, 5.5 mM) or HG (35 mM) for 48 h. Protein expression of iNOS and Arg1 was assessed by WB. β-actin served as a loading control. Quantitative analysis is shown in the right panels. Full-length uncropped blots are shown in Supplementary Figure S16. (**G**) RAW 264.7 cells were transfected with either negative control siRNA (si-NC) or ID3-specific siRNA (si-ID3), followed by treatment with HG (35 mM) alone or in combination with TNF-α&IFN-γ-Exo (10 μg/ml) for 48 h. The mRNA expression levels of the M1 marker iNOS and the M2 marker Arg1 were assessed by qRT-PCR and normalized to β-actin. (**H**) RAW 264.7 cells were treated as in panel (G). Protein levels of phosphorylated p65 (p-p65) and total p65 (t-p65) were assessed by WB after 1-2 h of treatment. β-actin served as a loading control. The p-p65/t-p65 ratio was quantified and normalized to the NC + HG group. Full-length uncropped blots are shown in Supplementary Figure S17. (*n* = 3), statistical analysis was performed using one-way ANOVA followed by Tukey’s post hoc test, ns indicates no significant difference, *P* > 0.05, *P < 0.05, **P < 0.01, ***P < 0.001, ****P < 0.0001.

Comprehensive analysis of UID RNA-seq data revealed significant upregulation of CD5L, Parp16, and Neurog2 genes, which are functionally associated with immune regulation, inflammatory response, and cellular differentiation. Conversely, we observed marked downregulation of ID3, DMPK, and Gbp7 genes, which participate in transcriptional regulation, cell differentiation, and innate immunity processes. Subsequent validation by qRT-PCR (Supplementary Figure S5A) and WB (Figure 6A) confirmed that ID3 expression was elevated in the HG group but significantly reduced following treatment with both Norm-Exo and TNF-α&IFN-γ-Exo.

To investigate ID3′s role in MSC-Exo-mediated macrophage polarization, we employed an ID3 overexpression plasmid, which demonstrated high transfection efficiency (Figure 6B) and was subsequently used for functional studies. Comparative analysis revealed that the ID3 overexpression group showed comparable protein expression levels of ID3, iNOS, and Arg1 to both Vehicle + HG and ID3 + HG groups (*P* > 0.05), with the polarization effects mirroring those induced by high glucose stimulation (Figure 6C). This observation suggests that ID3 participates in macrophage polarization under hyperglycemic conditions.

Notably, when compared to the ID3 overexpression group, both ID3 + Exo and ID3 + TNF-α&IFN-γ-Exo groups exhibited reduced iNOS expression. While Arg1 expression was significantly elevated in the ID3 + TNF-α&IFN-γ-Exo group (*P* < 0.05), no significant change was observed in the ID3 + Exo group (*P* > 0.05) (Figure 6D). These findings demonstrate that both MSC-Exo preparations similarly influence macrophage polarization markers under high glucose conditions, with ID3 emerging as a potential key regulatory target.

Further comparative analysis demonstrated that the inhibitory effect on iNOS expression and the stimulatory effect on Arg1 expression were substantially attenuated in both HG + ID3+Exo and HG + ID3+TNF-α&IFN-γ-Exo groups when compared to their respective non-ID3 overexpressing counterparts (HG + Exo and HG + TNF-α&IFN-γ-Exo groups) (Figure 6E). These results collectively indicate that suppression of ID3 expression participates in the regulation of macrophage polarization by MSC-derived exosomes in hyperglycemic environments.

To further investigate whether ID3 mediates the effects of MSC-Exo on macrophage polarization, we performed ID3 knockdown experiments. As shown in Figure 6F, high glucose treatment (NC + HG) significantly upregulated iNOS and downregulated Arg1 compared with the normal glucose control (NC + NG). ID3 knockdown alone under high glucose conditions (siID3 + HG) effectively reversed this phenotype, reducing iNOS and increasing Arg1 expression. Notably, ID3 knockdown under normal glucose conditions (siID3 + NG) did not alter iNOS or Arg1 levels, indicating that ID3 specifically regulates polarization under hyperglycemic stress.

To determine whether exosome effects depend on ID3, we compared the effects of TNF-α&IFN-γ-Exo in control and ID3-knockdown cells. qRT-PCR analysis (Figure 6G) revealed that TNF-α&IFN-γ-Exo treatment significantly reduced iNOS and increased Arg1 mRNA levels in control cells (NC + HG+ TNF-α&IFN-γ-Exo vs. NC + HG). ID3 knockdown alone (si-ID3 + HG) produced a similar effect. Importantly, when ID3 was knocked down, additional TNF-α&IFN-γ-Exo treatment (si-ID3 + HG+ TNF-α&IFN-γ-Exo) still induced a further, albeit attenuated, improvement compared with si-ID3 + HG alone, suggesting that while ID3 is a critical mediator, additional ID3-independent pathways also contribute to the full effect of exosomes.

We next examined whether NF-κB signaling is involved in this process. As shown in Figure 6H, high glucose increased p-p65 levels, which was attenuated by either ID3 knockdown or TNF-α&IFN-γ-Exo treatment. Notably, when ID3 was knocked down, the inhibitory effect of TNF-α&IFN-γ-Exo on p-p65 was partially but not completely abrogated, consistent with the partial dependence of exosome action on ID3.

To address the limitation of using an immortalized murine macrophage cell line and to enhance translational relevance, we further validated the role of ID3 in a human macrophage model. THP-1 cells were differentiated into adherent macrophages using PMA and subjected to siRNA-mediated knockdown. Among three candidate siRNAs, ID3-213 exhibited the highest knockdown efficiency and was selected for subsequent experiments (Supplementary Figure S6A). Under high glucose conditions, THP-1-derived macrophages showed a significant increase in pro-inflammatory cytokines (TNF-α and IL-6) and a decrease in anti-inflammatory markers (CD206 and ARG1) compared with the normal glucose group. Importantly, ID3 knockdown markedly attenuated the expression of TNF-α and IL-6 while restoring CD206 and ARG1 levels under high glucose conditions (Supplementary Figure S6B), supporting an ID3-dependent regulation of macrophage polarization.

**Figure 7:**
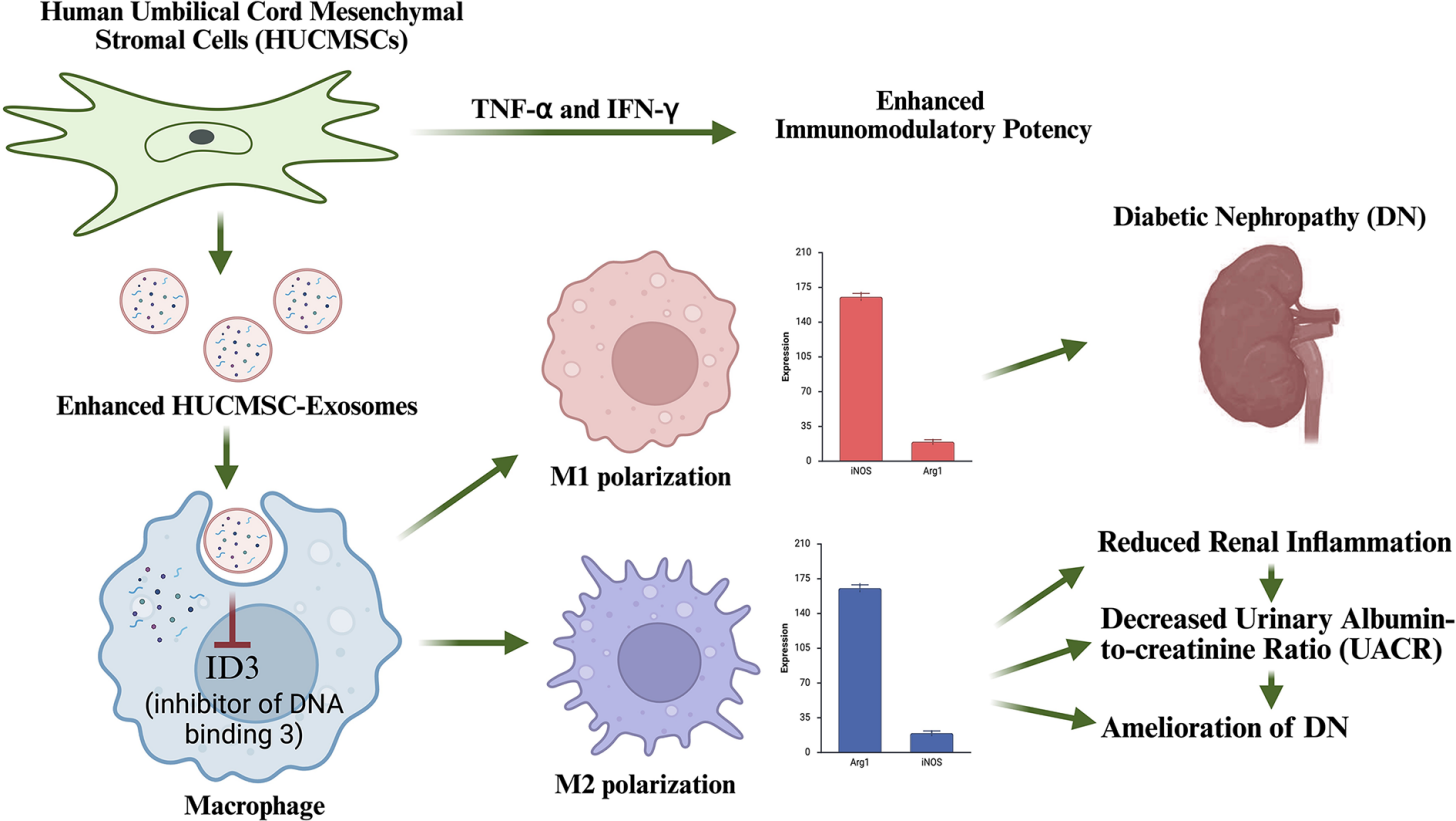
Schematic illustration of the proposed mechanism by which TNF-α&IFN-γ-pretreated MSC-derived exosomes regulate macrophage polarization Exosomes are taken up by macrophages, leading to suppression of ID3 expression, thereby promoting a shift from pro-inflammatory M1 to anti-inflammatory M2 phenotype. This ultimately results in reduced renal inflammation and improved outcomes in DN. Figure created in Biorender.com.

Based on these findings, we propose a working model summarizing the mechanism by which TNF-α&IFN-γ-pretreated MSC-derived exosomes regulate macrophage polarization to attenuate diabetic renal injury (Figure 7).

## Discussion

DM is a chronic metabolic disorder characterized by persistent hyperglycemia, which can lead to multiple severe complications. DN represents the most critical microvascular complication of DM and serves as the primary cause of ESRD [33,34]. Current understanding positions DN as an inflammatory disease, with chronic inflammation recognized as a pivotal factor in its progression [14,35]. MSCs demonstrate considerable therapeutic potential for inflammatory-related diseases due to their potent immunomodulatory properties, exhibiting tissue repair capabilities across various inflammatory animal models [36–39]. Notably, MSCs have shown renoprotective effects in DN mouse models [40]. Theoretically, harnessing the immunomodulatory effects of MSC-Exo to mitigate inflammatory responses in DN and achieve renal protection appears feasible. Furthermore, pretreatment of MSC-Exo with inflammatory factors may enhance these therapeutic outcomes [24].

In our *in vivo* experiments, we established an STZ-induced T1DM mouse model. At 12 weeks post-induction, diabetic mice received tail vein injections of either Norm-Exo or TNF-α&IFN-γ-Exo. Our results demonstrated that both exosome preparations effectively reduced blood glucose and UACR levels, improved glucose tolerance, ameliorated systemic inflammatory responses, alleviated local renal inflammation, attenuated kidney damage, and suppressed the secretion of various pro-inflammatory cytokines. Flow cytometric analysis of splenic immune cells revealed that both Norm-Exo and TNF-α&IFN-γ-Exo reduced the proportions of Th1 and Th17 cells while increasing Th2 and Treg cell populations, with TNF-α&IFN-γ-Exo exhibiting more pronounced effects. Additionally, both exosome treatments decreased splenic monocyte percentages, indicating their capacity to modulate systemic inflammation in diabetic mice. Importantly, kidney tissue analysis showed decreased levels of IL-6, IL-1β, and TNF-α alongside elevated IL-10 in exosome-treated groups. Flow cytometry of renal single-cell suspensions demonstrated reduced CD86^+^ (M1 phenotype) macrophages and increased CD206^+^ (M2 phenotype) macrophages following exosome treatment. Given the crucial role of macrophages in renal inflammation [12], we propose that the renoprotective effects of Norm-Exo and TNF-α&IFN-γ-Exo may be mediated through regulation of macrophage polarization. Notably, correlation analyses revealed strong associations between glycaemic indices and renal injury as well as inflammatory markers, suggesting that improved glycaemic control may contribute to the observed renoprotective effects. However, given the direct immunomodulatory effects of exosomes on macrophage polarization observed *in vitro*, it is likely that both glucose-dependent and glucose-independent mechanisms are involved. Importantly, the observed immunological alterations were interpreted relative to non-diabetic baseline values, demonstrating that exosome treatment not only modulates inflammatory responses but also partially restores immune homeostasis toward physiological conditions.

It is worth noting that both Norm-Exo and TNF-α&IFN-γ-Exo significantly reduced blood glucose levels in STZ-induced diabetic mice. However, the present study was not designed to elucidate the mechanisms underlying glucose lowering. Considering the well-established immunomodulatory properties of mesenchymal stem-cell-derived exosomes [41–43], we speculate that the observed improvement in glycaemic control is more likely secondary to their anti-inflammatory effects. Specifically, exosomes may improve systemic metabolic homeostasis and insulin sensitivity by alleviating chronic inflammation [30,32,44,45], rather than through direct protection of pancreatic β cells or direct reversal of insulin resistance. Therefore, the glucose-lowering effect should be regarded as a secondary benefit, whereas attenuation of renal inflammation and regulation of macrophage polarization represent the primary mechanisms by which exosomes confer protection against DN.

In our *in vitro* experiments, we established a high glucose model using RAW264.7 cells to elucidate the potential mechanisms by which Norm-Exo and TNF-α&IFN-γ-Exo regulate macrophage polarization. During our investigation of the optimal duration for hyperglycemic stimulation, we observed a significant increase in M2 macrophages after 24 h of exposure to high glucose conditions, followed by a subsequent decrease at 48 h. This temporal pattern may be explained by the initial compensatory increase in anti-inflammatory M2 macrophages during the early phase (24 h) of hyperglycemia-induced inflammatory response, which serves to counteract the developing inflammation. However, as the inflammatory response progresses and the M1/M2 balance shifts, we observed an increase in pro-inflammatory M1 macrophages accompanied by a corresponding decrease in anti-inflammatory M2 macrophages at the later time point (48 h).

Importantly, the use of an iso-osmotic mannitol control confirmed that the observed effects were glucose-specific rather than driven by osmotic stress. Consistent with our *in vivo* findings, loss-of-function experiments in RAW 264.7 cells revealed that ID3 knockdown alone significantly reduced iNOS and increased Arg1 expression under high glucose conditions, phenocopying the effect of exosome treatment. Moreover, when ID3 was knocked down, the additional effect of TNF-α&IFN-γ-Exo on iNOS and Arg1 mRNA expression was attenuated, although a partial improvement remained. These results indicate that ID3 is a critical, but not exclusive, mediator of exosome action. Furthermore, we observed that both ID3 knockdown and exosome treatment suppressed high glucose-induced p-p65 activation, and the inhibitory effect of exosomes was partially dependent on ID3. This suggests that the ID3-mediated transcriptional axis converges, at least in part, on the NF-κB signaling pathway.

To enhance translational relevance, we further validated our findings in PMA-differentiated THP-1 macrophages. Under high glucose conditions, THP-1-derived macrophages exhibited a significant increase in pro-inflammatory cytokines (TNF-α and IL-6) and a decrease in anti-inflammatory markers (CD206 and ARG1), confirming the establishment of an M1-skewed model. Importantly, ID3 knockdown markedly attenuated TNF-α and IL-6 expression while restoring CD206 and ARG1 levels. These results support a conserved role of ID3 in regulating macrophage polarization across species, thereby increasing the translational relevance of our findings.

Through comprehensive transcriptome sequencing analysis, we systematically examined the gene expression changes associated with macrophage polarization under the influence of Norm-Exo and TNF-α&IFN-γ-Exo. The key genes identified through this approach were subsequently validated using both qRT-PCR and WB analyses. Furthermore, we employed plasmid transfection technology to overexpress these candidate genes, allowing us to confirm their functional roles in the macrophage polarization process. Our GO enrichment and KEGG pathway analyses of DEGs revealed a particularly strong association between ID3 and macrophage polarization. ID3, a transcription regulatory factor with broad cellular expression, has been previously implicated in the regulation of various biological processes including inflammation, immune responses, and cell proliferation [46–48].

To specifically examine the effects of Norm-Exo and TNF-α&IFN-γ-Exo on ID3 expression under high glucose conditions, we performed qRT-PCR and WB analyses to quantify ID3 mRNA and protein levels across treatment groups. These experiments demonstrated that both exosome preparations significantly downregulated ID3 expression compared to the high glucose model group, with TNF-α&IFN-γ-Exo exhibiting a more pronounced effect. These findings were consistent with our transcriptome sequencing results and suggest that ID3 may mediate the regulatory effects of extracellular vesicles on macrophage behavior.

To further investigate the functional importance of ID3 in macrophage regulation under high glucose conditions, we performed ID3 overexpression experiments. Our results showed no significant differences in ID3, iNOS, and Arg1 protein expression levels between the ID3 overexpression group and either the Vehicle + HG or ID3 + HG control groups (*P* > 0.05), indicating that ID3 overexpression alone produces effects on macrophage polarization markers similar to those induced by high glucose conditions. Subsequent experiments involving treatment of ID3-overexpressing RAW264.7 cells with Norm-Exo and TNF-α&IFN-γ-Exo demonstrated that both exosome preparations significantly inhibited M1 polarization, while TNF-α&IFN-γ-Exo showed additional capacity to promote M2 polarization. These observations mirror the effects of MSC-Exo on macrophage polarization under high glucose conditions and suggest that ID3 participates in this regulatory process.

Distinct from previously reported immunomodulatory mechanisms of MSC-derived exosomes (MSC-Exo), the ID3-mediated transcriptional regulatory pathway identified in this study represents a novel mechanism. Conventional studies have largely attributed the effects of MSC-Exo to non-coding RNAs or intracellular signaling mediators [49,50]. In contrast, our findings demonstrate that ID3 functions as an upstream transcriptional regulator involved in exosome-mediated macrophage polarization toward the M2 phenotype. Importantly, this mechanism was validated under HG conditions relevant to DN, thereby more closely reflecting the pathological microenvironment and enhancing the translational relevance of our findings. Notably, the ID3-mediated transcriptional axis appears to operate, at least in part, independently of classical inflammatory signaling pathways, providing an additional mechanistic layer to explain the therapeutic effects of MSC-Exo in DN.

Furthermore, regulation of splenic CD4^+^ T cell subsets and renal macrophage polarization should not be viewed as independent processes. Exosomes can modulate systemic immune responses by reshaping T cell subsets and alleviating systemic inflammation [51,52], which may indirectly favor macrophage polarization toward the M2 phenotype in the kidney. In parallel, our data indicate that exosomes can directly regulate macrophage polarization via the ID3-dependent pathway. These systemic and local immunomodulatory effects likely act in concert to mitigate renal injury in DN.

Additional confirmation came from experiments where we overexpressed ID3 in the presence of exosome treatments. Under these conditions, we observed a gradual attenuation of the exosomes’ ability to inhibit M1 polarization and promote M2 polarization, providing evidence that suppression of ID3 expression contributes to the regulation of macrophage polarization by extracellular vesicles. The current study has systematically explored the therapeutic potential of Norm-Exo and TNF-α&IFN-γ-Exo in DN, with particular emphasis on their capacity to remodel macrophage polarization. Our identification of ID3 as a molecule involved in this process represents a significant contribution to understanding the mechanisms underlying HUCMSC-Exo-mediated macrophage reprogramming in DN.

## Conclusions

Our findings demonstrate that both Norm-Exo and TNF-α&IFN-γ-Exo effectively reduced blood glucose levels and UACR in diabetic mice, with TNF-α&IFN-γ-Exo exhibiting superior therapeutic efficacy compared to Norm-Exo. Furthermore, both exosome preparations significantly ameliorated renal inflammatory responses in the diabetic mouse model, with TNF-α&IFN-γ-Exo again demonstrating more pronounced anti-inflammatory effects. Mechanistic investigations revealed that CD4^+^ T cells, monocytes, and macrophages collectively contribute to the regulation of renal inflammatory responses by Norm-Exo and TNF-α&IFN-γ-Exo in diabetic mice. Importantly, our study identified ID3 as a molecule that participates in exosome-mediated regulation of macrophage polarization under high glucose conditions. The experimental evidence suggests that Norm-Exo and TNF-α&IFN-γ-Exo likely modulate macrophage polarization in hyperglycemic environments, in part by suppressing ID3 expression, thereby exerting their therapeutic effects on DN.

## Clinical perspectives

- **Background:** DN is driven by chronic inflammation and macrophage polarization, and effective immunomodulatory therapies are urgently needed.
- **Results:** Exosomes derived from HUCMSCs, especially those pretreated with TNF-α and IFN-γ, alleviated renal injury in diabetic mice by suppressing ID3 expression and promoting M2 macrophage polarization.
- **Significance:** These findings identify the MSC-Exo–ID3–macrophage axis as a novel therapeutic target and support the translational potential of exosome-based, cell-free therapies for DN.

## Supplementary Material

Supplementary Figures S1-S17 and Tables S1-S3

Supplementary File Supplementary file_RNA

## Data Availability

The sequencing data supporting the results reported in our paper are archived in supplementary file named ‘Supplementary file_RNA sequencing data_All_Samples_rpkm.xls’. The datasets used and analyzed during the current study are available from the corresponding author (Lisha Li: lilisha@jlu.edu.cn) on reasonable request.

## Abbreviations

DEG: differentially expressed gene
DAB: 3,3‘-Diaminobenzidine
DM group: diabetes mellitus group
DM: diabetes mellitus
DM + Norm-Exo group: diabetes mellitus + Norm-Exo intervention group
DM + TNF-α&IFN-γ-Exo group: diabetes mellitus + pretreated MSC-Exo intervention group
DN: diabetic nephropathy
ESRD: end-stage renal disease
FDR: false discovery rate
GO: gene ontology
HE: hematoxylin and eosin
HG group: high-glucose group
HG + Norm-Exo group: high glucose + Norm-Exo intervention group
HG + TNF-α&IFN-γ-Exo group: high glucose + pre-treatment MSC-Exo intervention group
HUCMSCs: human umbilical cord mesenchymal stromal cells
H&E: hematoxylin and eosin
ID3: inhibitor of DNA binding 3
KEGG: Kyoto Encyclopedia of Genes and Genomes
M1: type 1 macrophages
M2: type 2 macrophages
MSC-Exo: mesenchymal stromal cell‐derived exosomes
MSCs: mesenchymal stromal cells
MTS: Masson’s trichrome stain
NC group: negative control group
Norm-Exo: normal MSC-Exo
NTA: nanoparticle tracking analysis
PAS: periodic acid-Schiff
PMA: Phorbol 12-Myristate 13-Acetate
qRT-PCR: quantitative real-time PCR
T1DM: type 1 diabetes
T2DM: type 2 diabetes
TEM: transmission electron microscopy
TNF-α&IFN-γ-Exo: TNF-α&IFN-γ pretreated MSC-Exo
UACR: urinary albumin-to-creatinine ratio
UID: unique identifier
UMI: unique molecular identifier
WB: western blot
