# Supplementary material for: Human umbilical cord mesenchymal stromal cell-derived exosomes from MSCs pretreated with inflammatory factors attenuate renal injury of diabetic mice by regulating macrophage polarization
Source: Clin Sci (Lond). 2026 Jun 10;140(7):1223–41. doi: 10.1042/CS20258827 (PMC13259867; doi:10.1042/CS20258827)
Supplement: Supplementary Figures S1-S17 and Tables S1-S3 [file CS-2025-8827_supp.pdf]

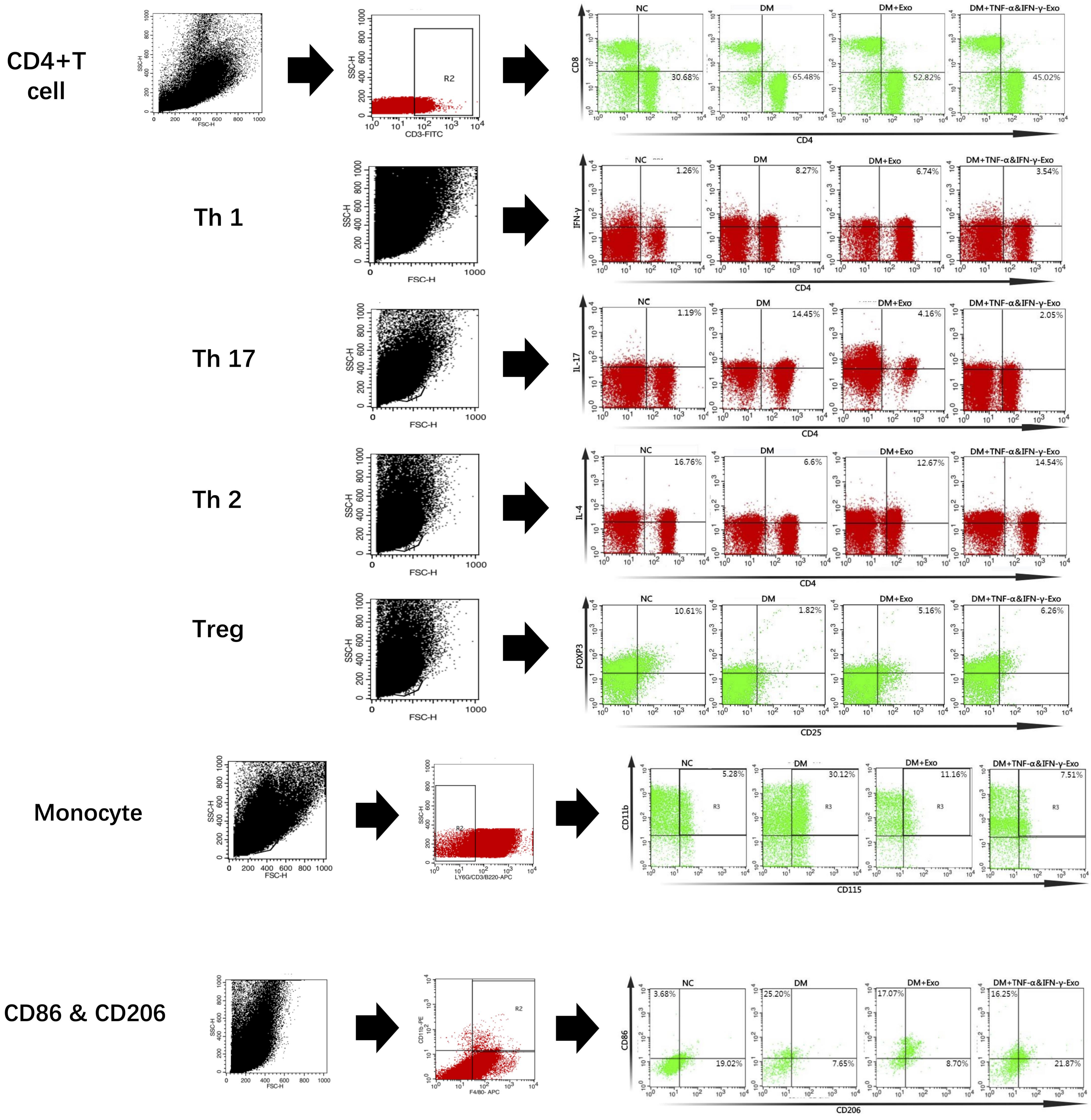

Supplementary Figure 1. Gating strategies for CD4^{+}T cells and their subsets (Th1, Th2, Th17, Treg, Monocyte, CD86&CD206)

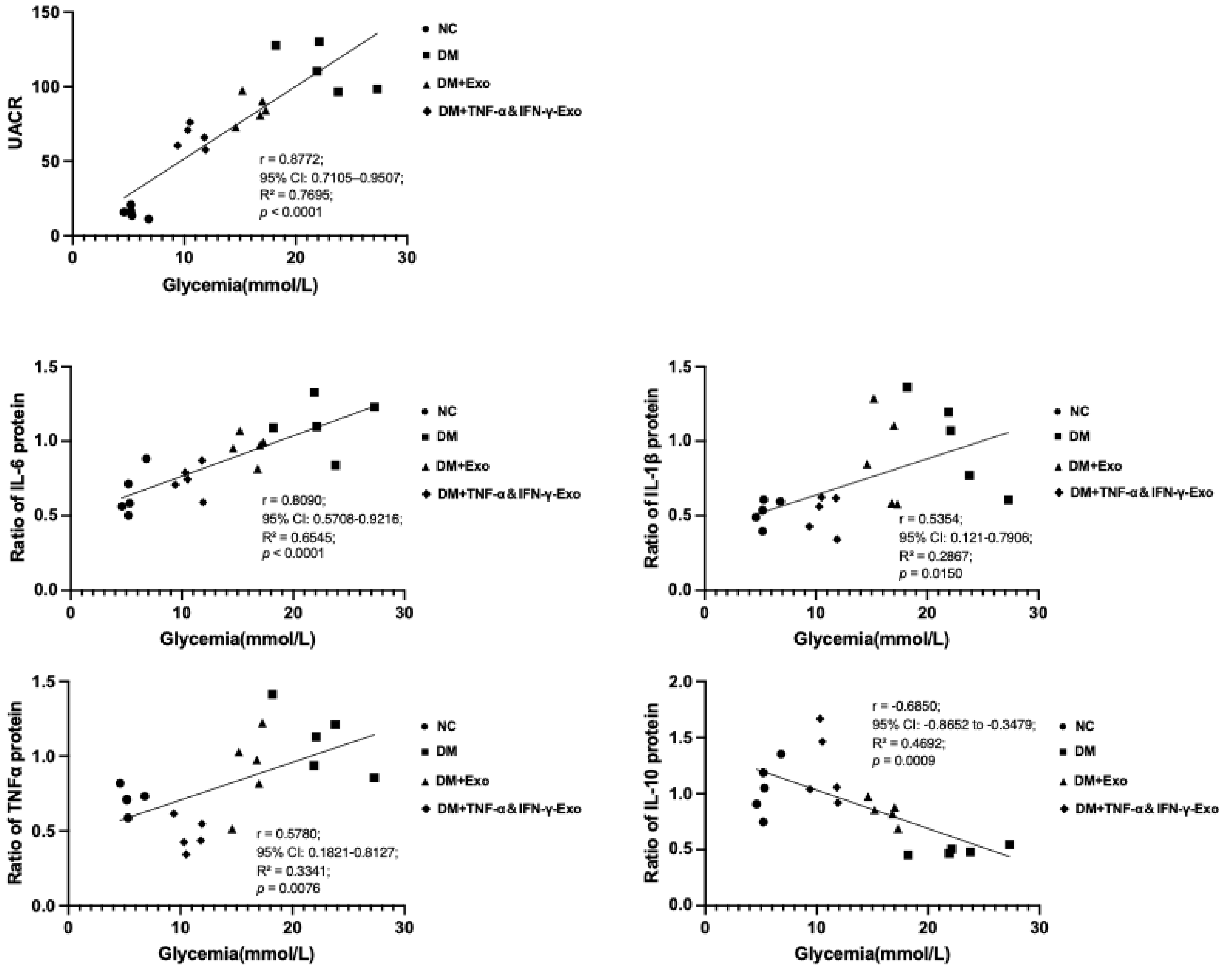

Supplementary Figure 2. Correlation between fasting blood glucose and renal injury or inflammatory markers.

Correlation analyses were performed between fasting blood glucose levels and UACR, IL-6, IL-1β, TNF-α, and IL-10. Fasting blood glucose levels were positively correlated with UACR and pro-inflammatory cytokines, and negatively correlated with IL-10, suggesting an association between glycaemic control and renal inflammation.

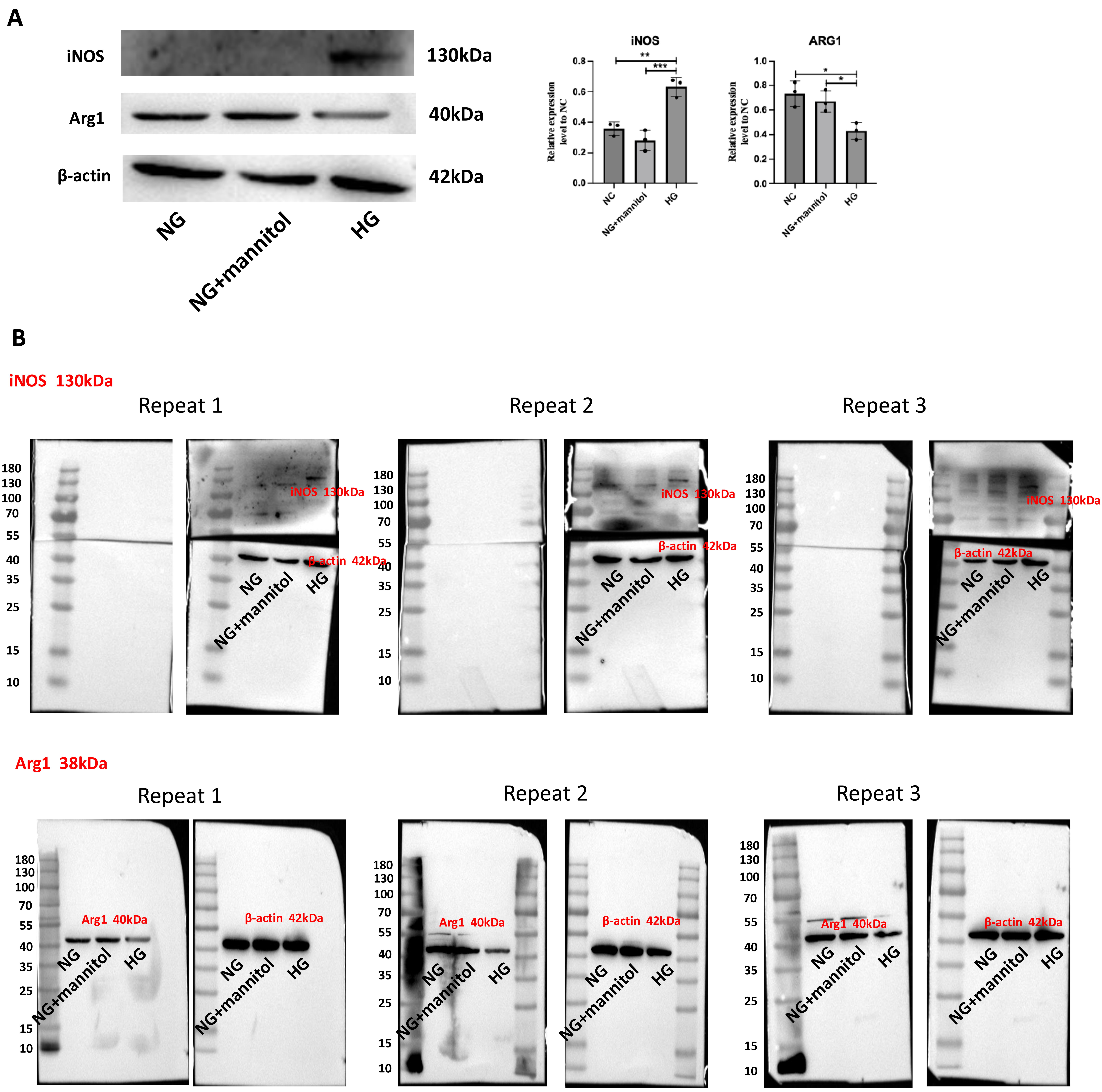

Supplementary Figure 3. Osmotic control using mannitol does not affect macrophage polarization.

A: RAW264.7 cells were cultured under normal glucose (NG, 5.5 mM), high glucose (HG, 35 mM), or iso-osmotic conditions (NG supplemented with 23.9 mM mannitol). Protein expression of iNOS and Arg1 was analyzed by western blot. Full-length uncropped blots are shown in B.

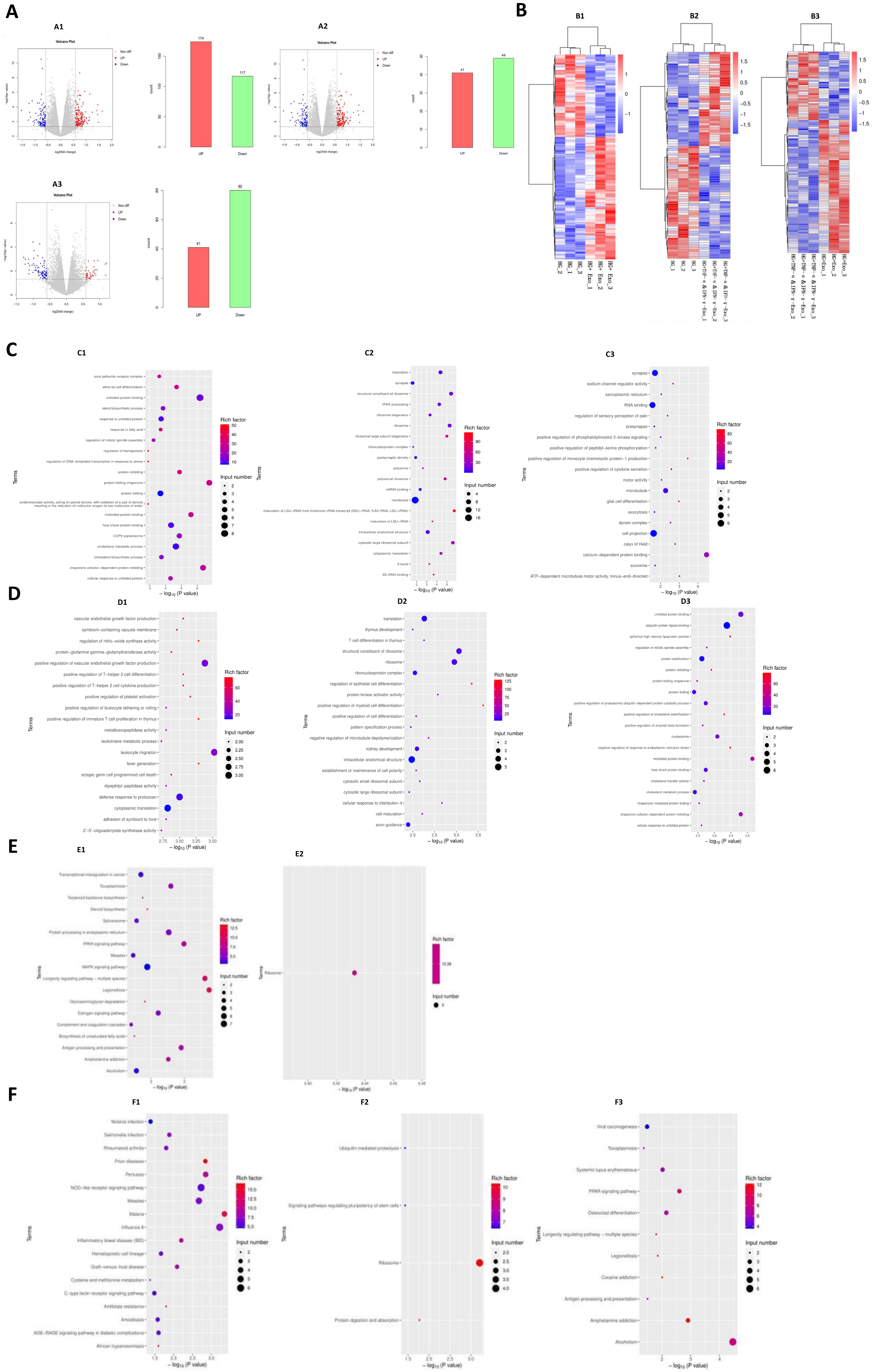

Supplementary Figure 4. UID RNA-seq reveals changes of HUCMSC-Exo on the mRNA expression profile in High-glucose-stimulated RAW264.7 cells. RAW 264.7 cells were stimulated by high glucose (35mmol/L glucose) for 48 h,followed by Norm-Exo (10 μg/mL) and TNF-α&IFN-γ-Exo (10 μg/mL) for 48 h. Samples from HG group, HG+Norm-Exo group and HG+TNF-α&IFN-γ-Exo group were collected for UID RNA-seq. A: Volcano plot of significantly differential genes in HG+Norm-Exo group compared with HG group (A1); HG+TNF-α&IFN-γ-Exo group compared with HG group (A2); HG+TNF-α&IFN-γ-Exo group compared with HG+Norm-Exo group (A3). B: Heat map of significantly differential genes in HG+Norm-Exo group compared with HG group (B1); HG+TNF-α&IFN-γ-Exo group compared with HG group (B2); HG+TNF-α&IFN-γ-Exo group compared with HG+Norm-Exo group (B3). C: GO enrichment analysis of Top 20 up-regulated differential genes in HG+Norm-Exo group compared with HG group (C1); HG+TNF-α&IFN-γ-Exo group compared with HG group (C2); HG+TNF-α&IFN-γ-Exo group compared with HG+Norm-Exo group (C3). D: GO enrichment analysis of Top 20 down-regulated differential genes in HG+Norm-Exo group compared with HG group (D1); HG+TNF-α&IFN-γ-Exo group compared with HG group (D2); HG+TNF-α&IFN-γ-Exo group compared with HG+Norm-Exo group (D3). E: KEGG pathway enrichment analysis of up-regulated signaling transduction in HG+Norm-Exo group compared with HG group (E1); HG+TNF-α&IFN-γ-Exo group compared with HG group (E2). F: KEGG pathway enrichment analysis of down-regulated signaling transduction in HG+Norm-Exo group compared with HG group (F1); HG+TNF-α&IFN-γ-Exo group compared with HG group (F2); HG+TNF-α&IFN-γ-Exo group compared with HG+Norm-Exo group (F3), (n=3).

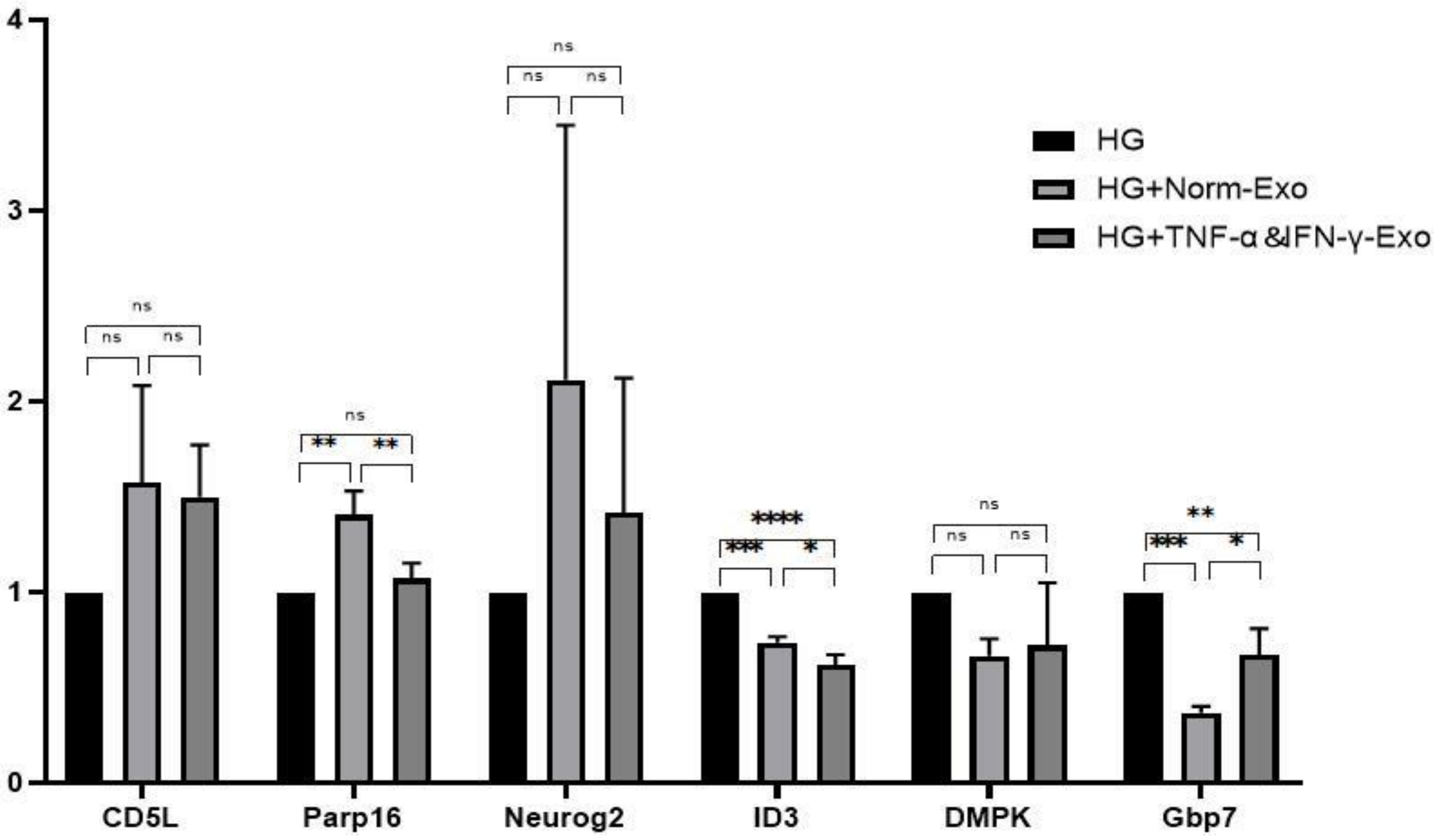

Supplementary Figure 5. ID3 was involved in -Exo mediated macrophage polarization.

qRT-PCR confirmed the expression of ID3 was upregulated in HG group and was downregulated after Norm-Exo and TNF-α&IFN-γ-Exo treatment.

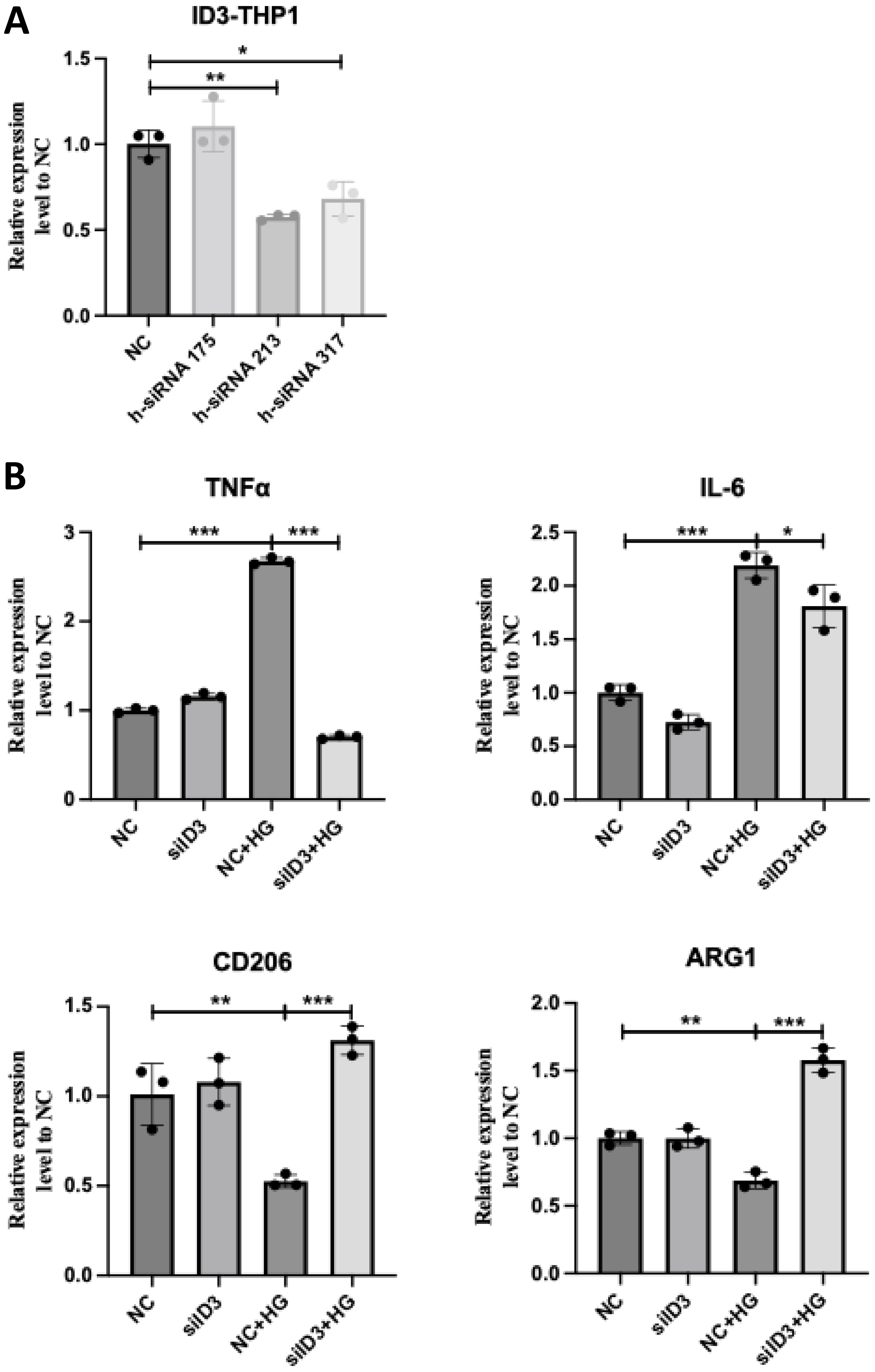

Supplementary Figure 6. ID3 knockdown regulates macrophage polarization in THP-1-derived macrophages.

(A) Knockdown efficiency of three candidate siRNAs targeting human ID3 (ID3-175, ID3-213, ID3-317) assessed by qRT-PCR. ID3-213 showed the highest efficiency and was selected for subsequent experiments.

(B) THP-1-derived macrophages were treated under normal glucose (NG) or high glucose (HG) conditions with or without ID3 knockdown. Expression of TNF-α, IL-6, CD206, and ARG1 was analyzed by qRT-PCR. High glucose increased pro-inflammatory markers and decreased anti-inflammatory markers, whereas ID3 knockdown reversed these effects. Data are presented as mean ± SD (n = 3). **P* < 0.05, ***P* < 0.01, ****P* < 0.001.

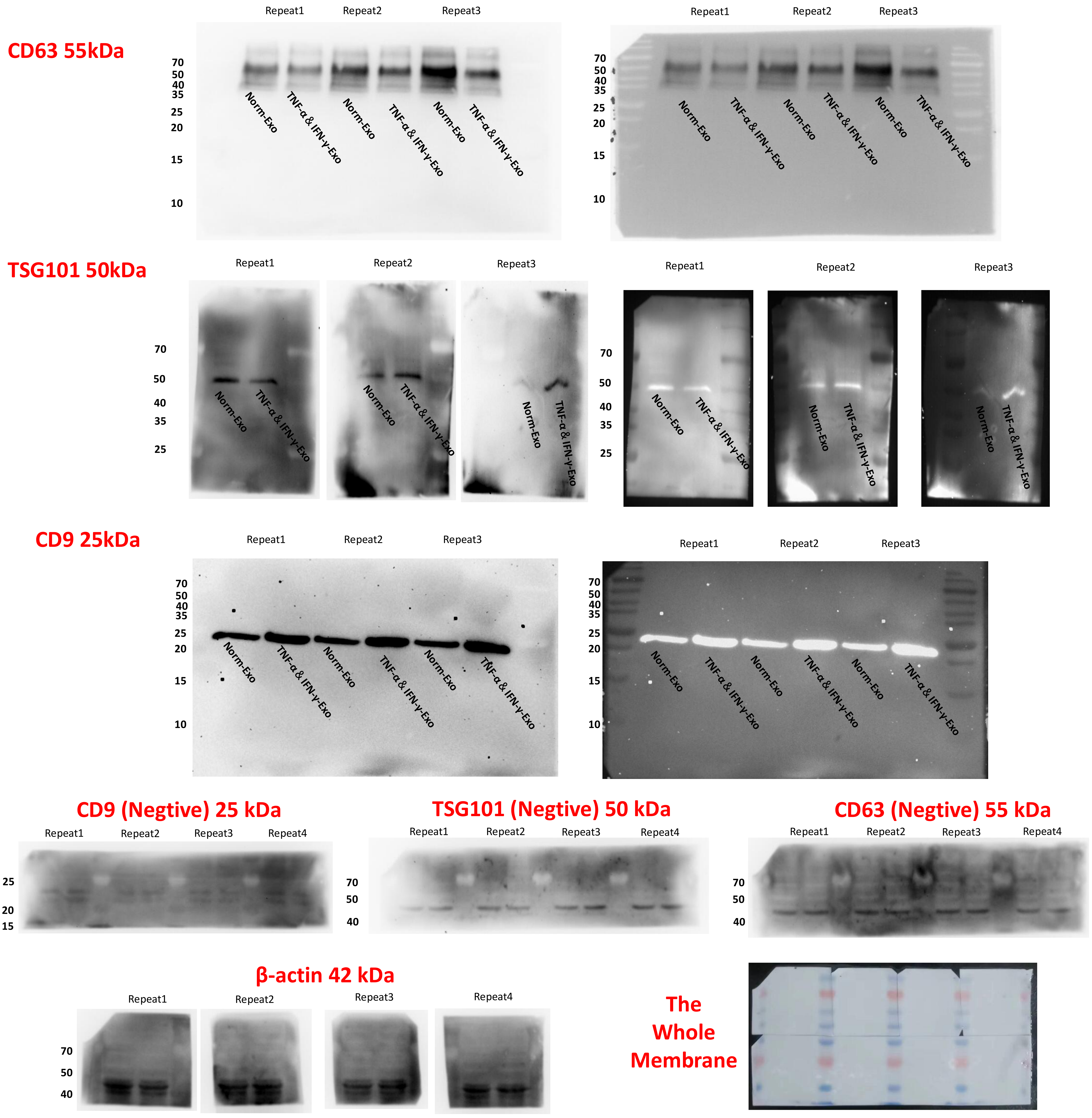

Supplementary Figure 7. WB confirmed that Norm-Exo and TNF-α＆IFN-γ-Exo express marker proteins CD9, CD63, and TSG101

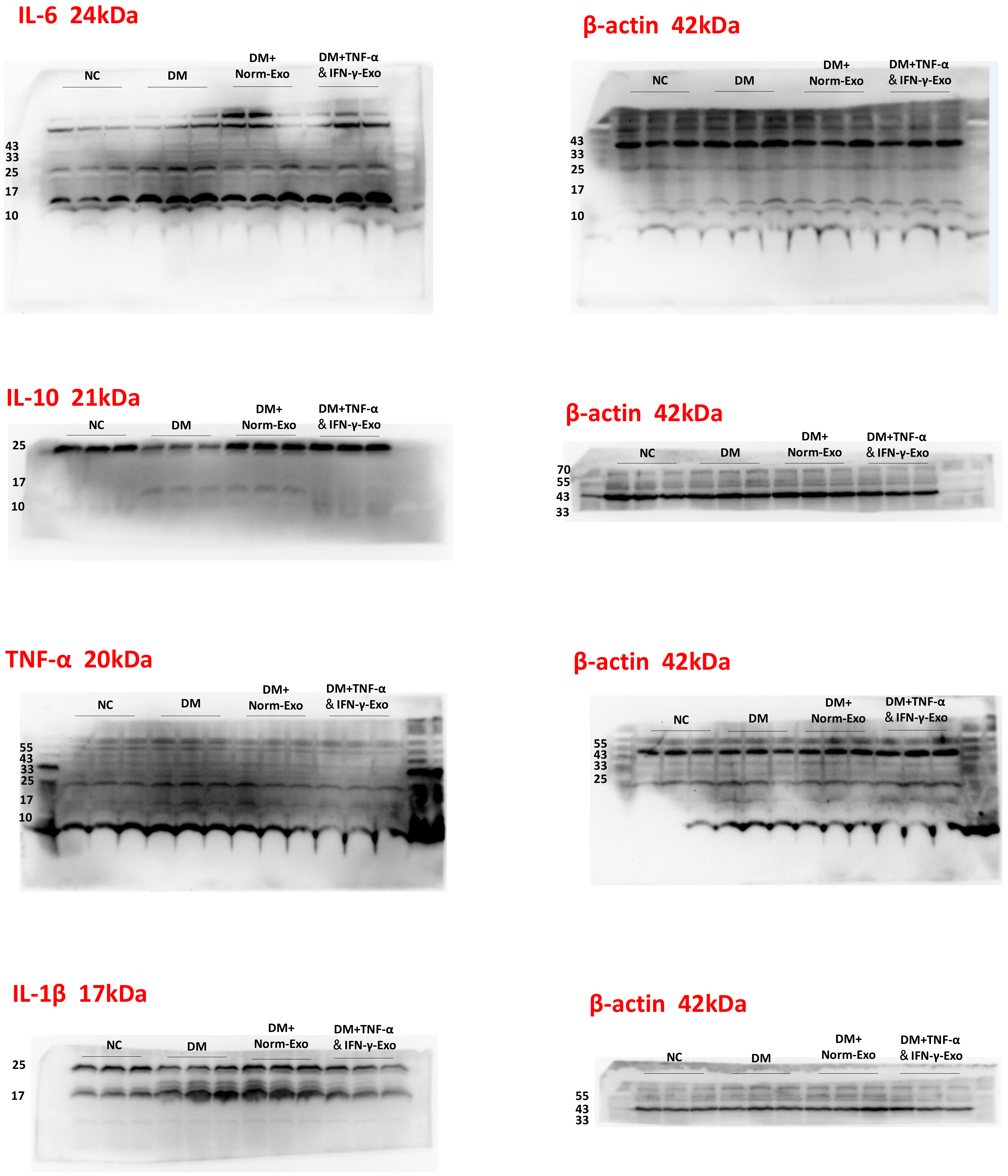

Supplementary Figure 8. WB and quantification results of IL-6, IL-1β, TNF-α and IL-10 in kidney tissue of mice

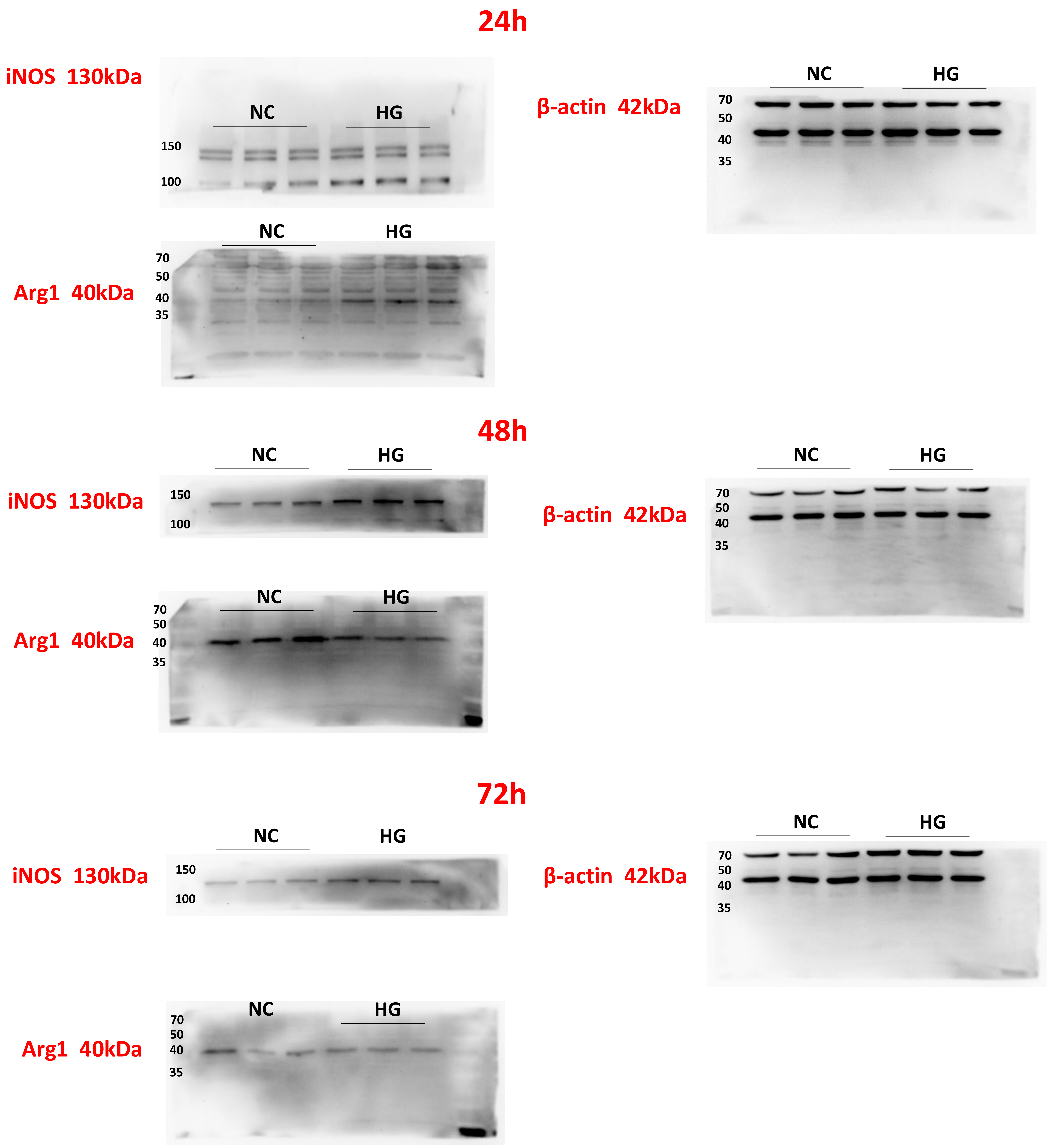

Supplementary Figure 9. WB and quantification results of iNOS and Arg1 in RAW264.7 cells cultured with high glucose (35 mmol/L) for 24h, 48h and 72h.

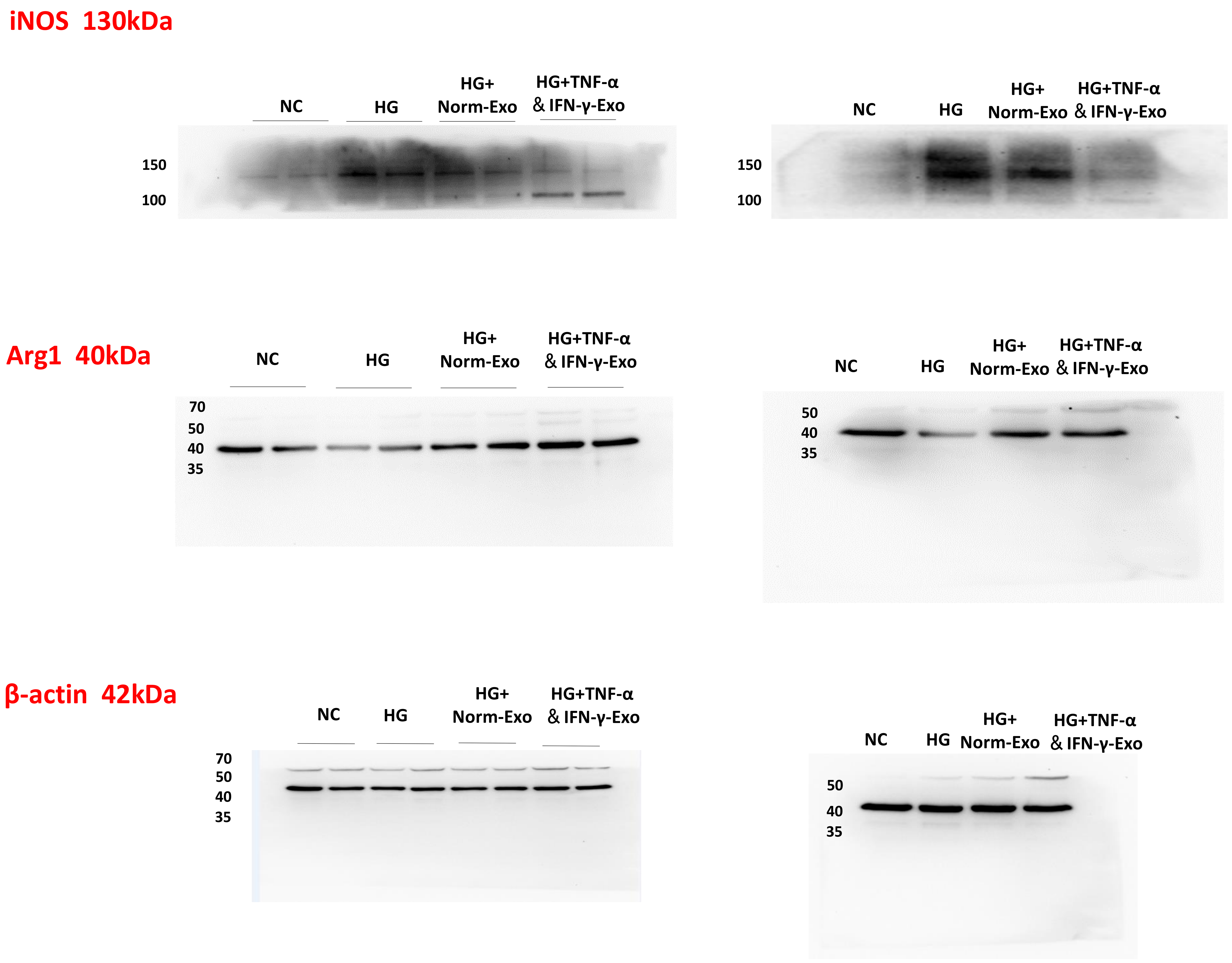

Supplementary Figure 10. WB and quantification results of iNOS and Arg1 in RAW264.7 cells

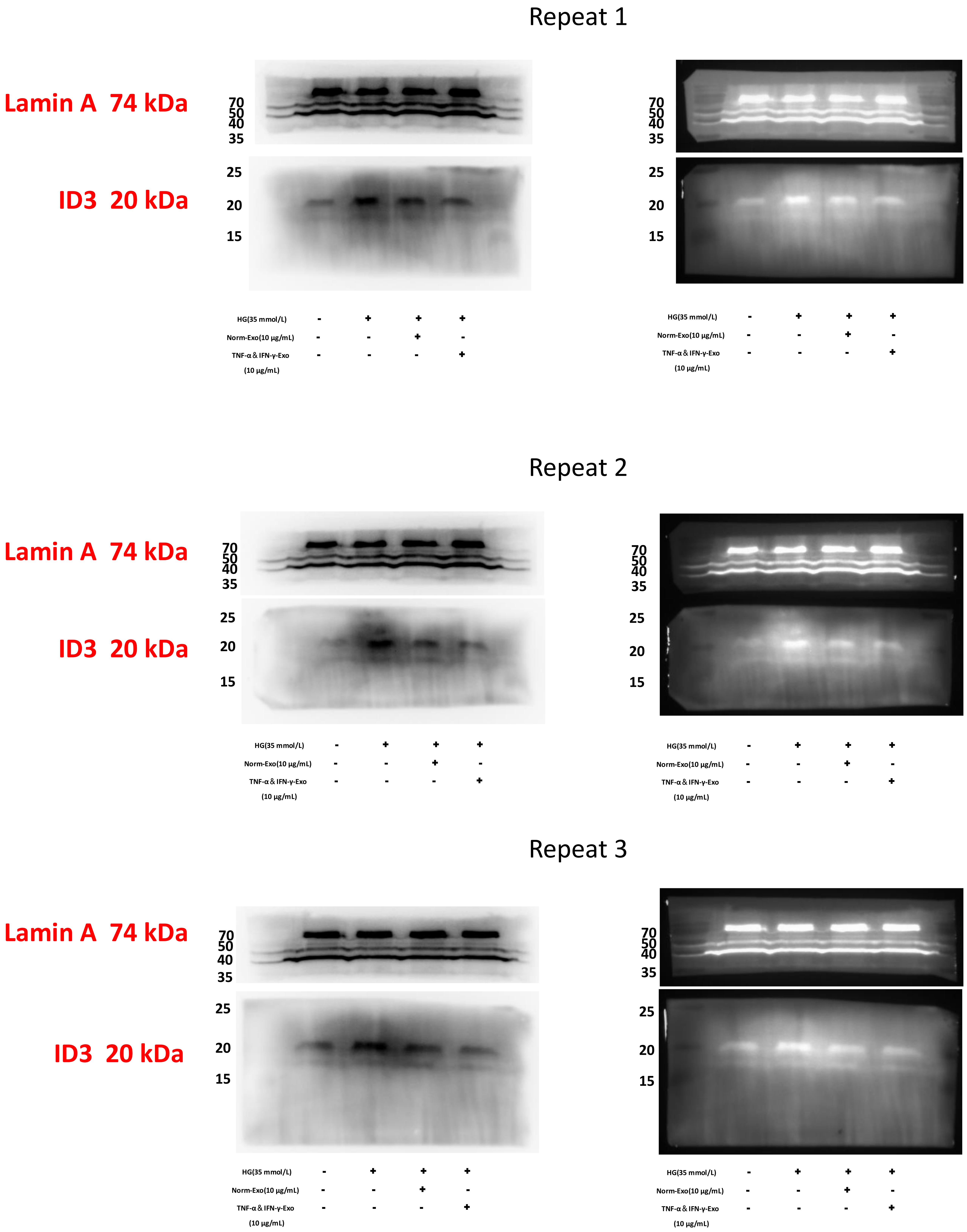

Supplementary Figure 11. WB confirmed the expression of ID3 was upregulated in HG group and was downregulated after Norm-Exo and TNF-α&IFN-γ-Exo treatment .

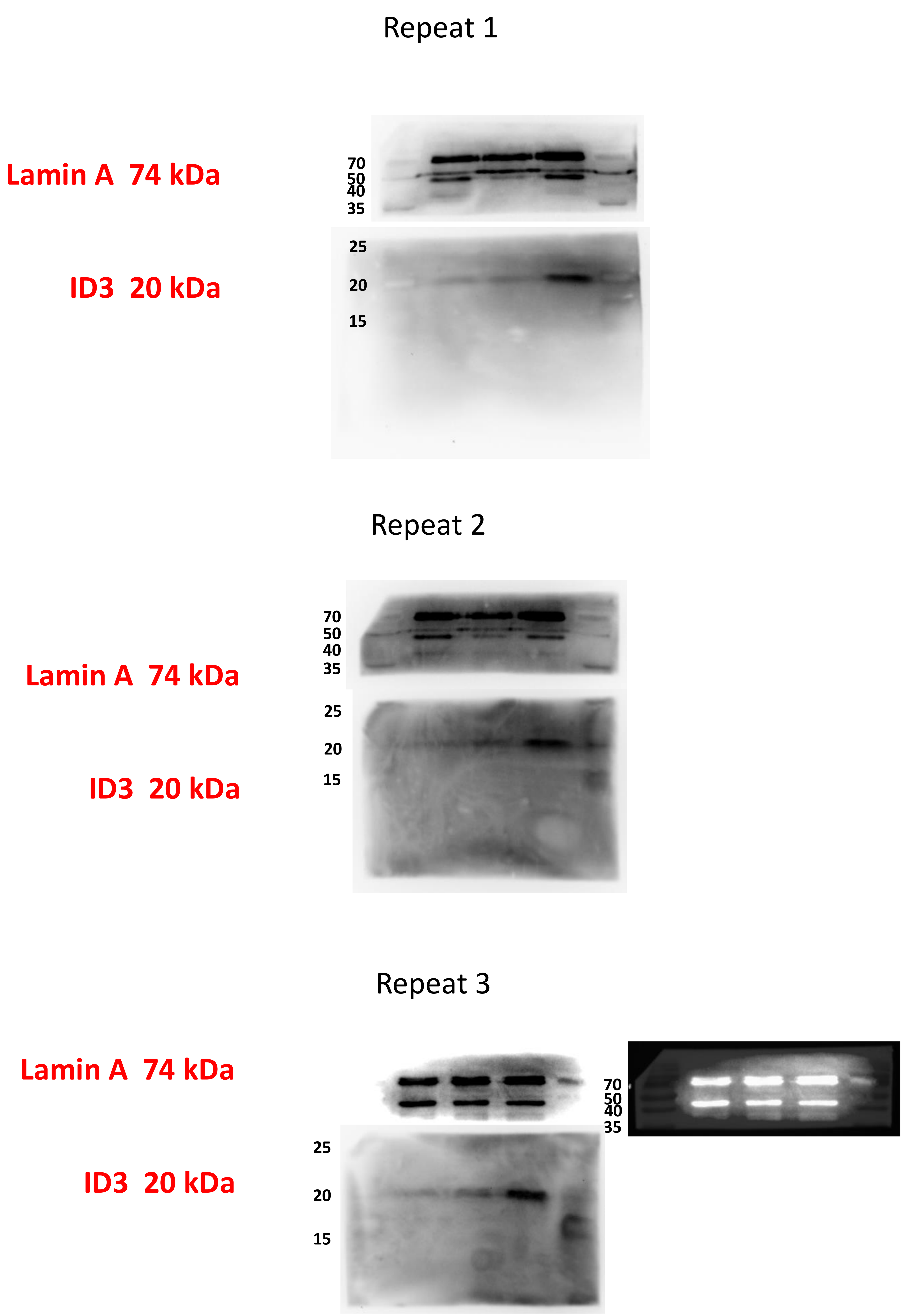

Supplementary Figure 12. ID3 overexpression transfection of RAW 264.7 cells. The expression level of ID3 was detected using WB and quantified by Image J

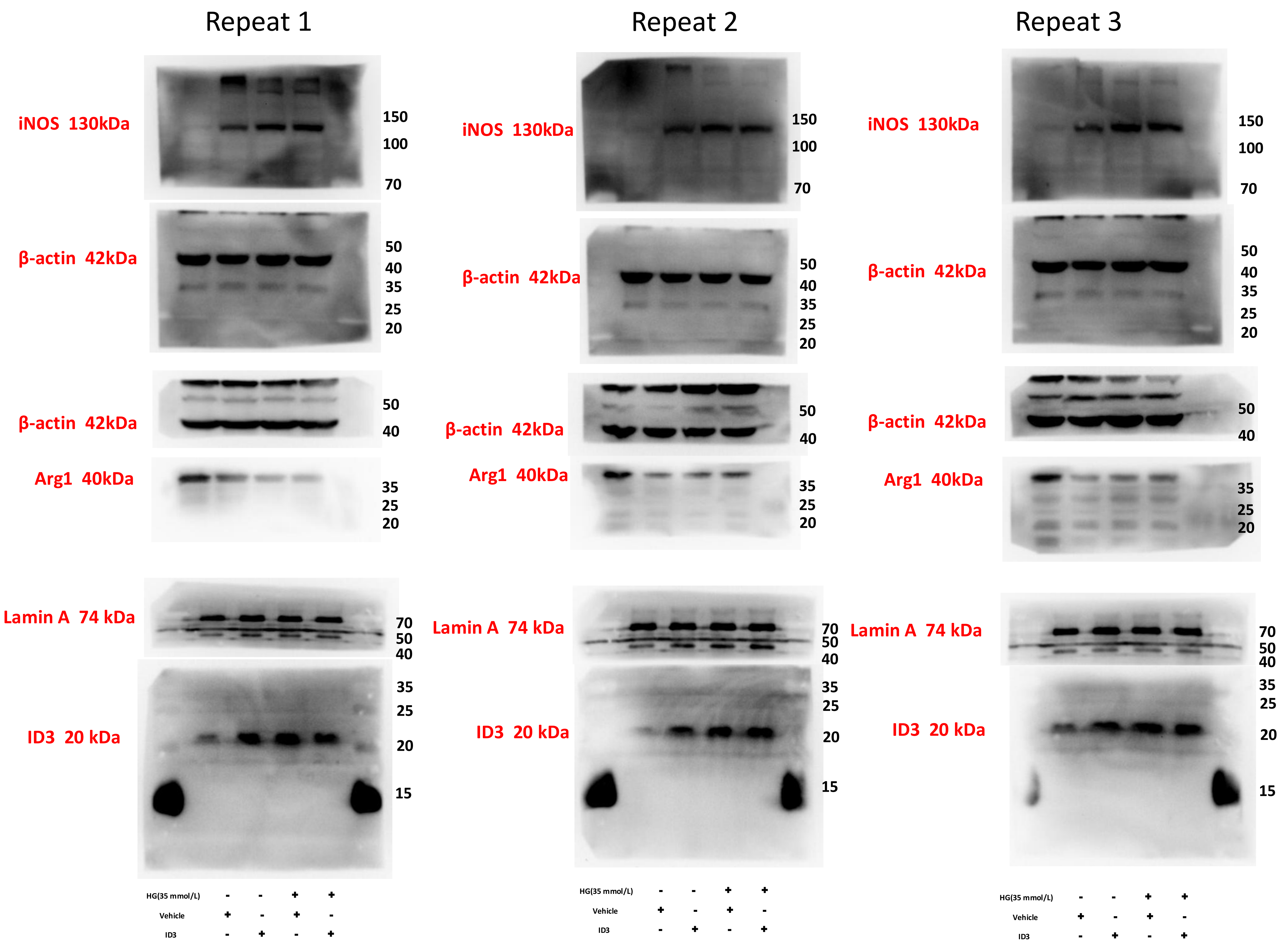

Supplementary Figure 13. After ID3 overexpression transfection of RAW 264.7 cells for 24h, followed by cultured with high glucose (35 mmol/L) for 48h. The expression level of ID3, iNOS and Arg1 was detected using WB

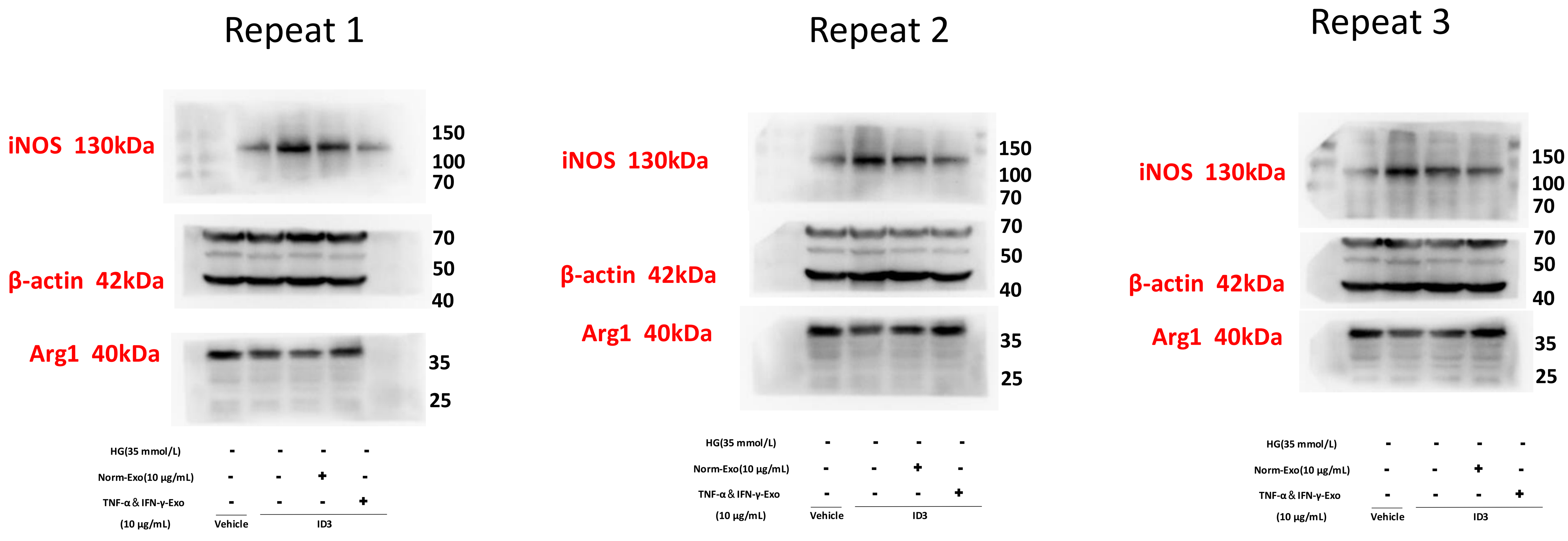

Supplementary Figure 14. After ID3 overexpression transfection of RAW 264.7 cells for 24h, followed by treatment with Norm-Exo and TNF-α&IFN-γ-Exo for 48h. The expression level of iNOS and Arg1 was detected using WB

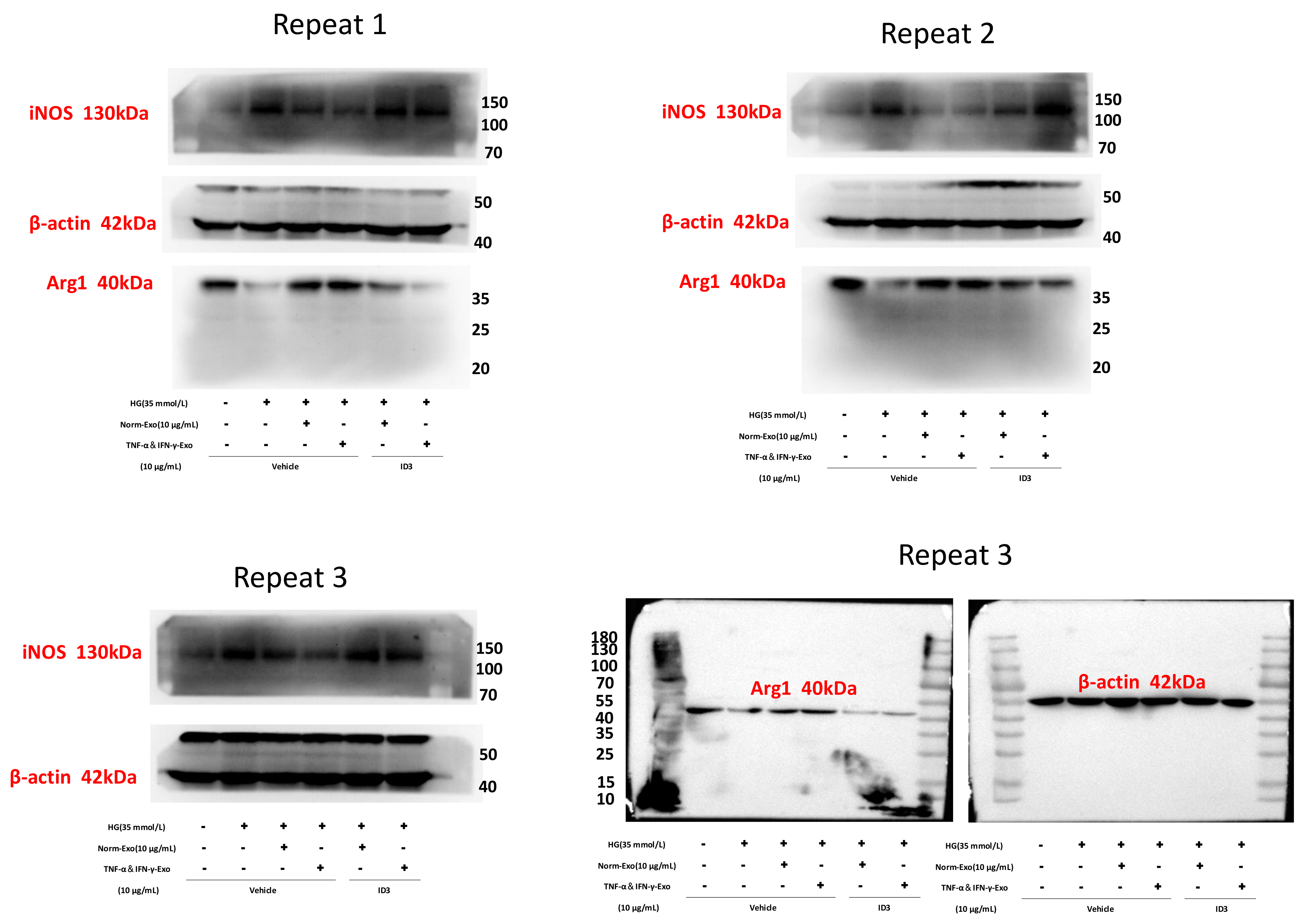

Supplementary Figure 15. After ID3 overexpression transfection of RAW 264.7 cells for 24h, followed by cultured with high glucose (35 mmol/L) for 48h, then treated with Norm-Exo and TNF-α&IFN-γ-Exo for 48h. The expression level of iNOS and Arg1 was detected using WB

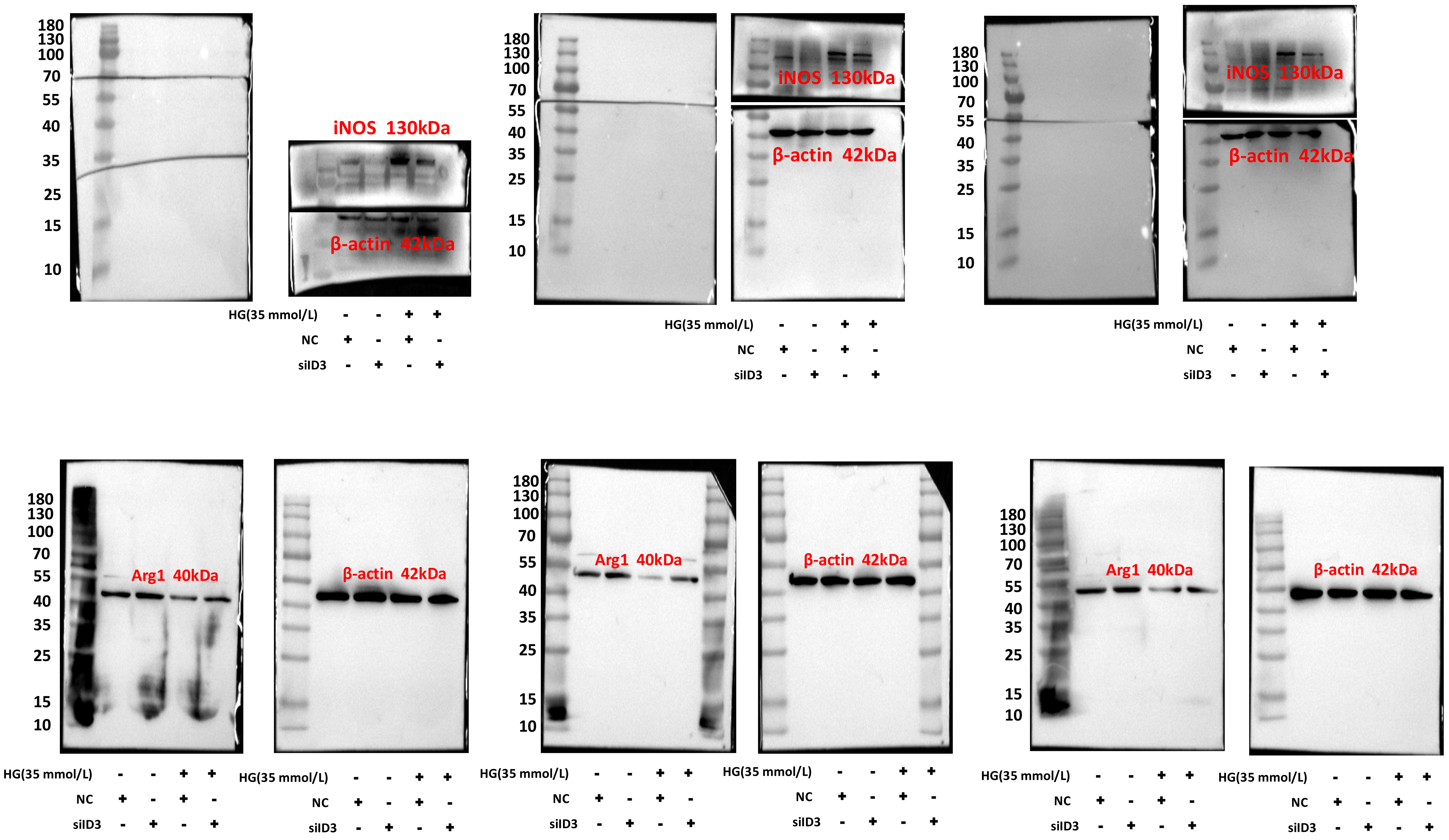

Supplementary Figure 16. After ID3 knockdown transfection of RAW 264.7 cells for 24h, followed by cultured with high glucose (35 mmol/L) for 48h. The expression level of iNOS and Arg1 was detected using WB.

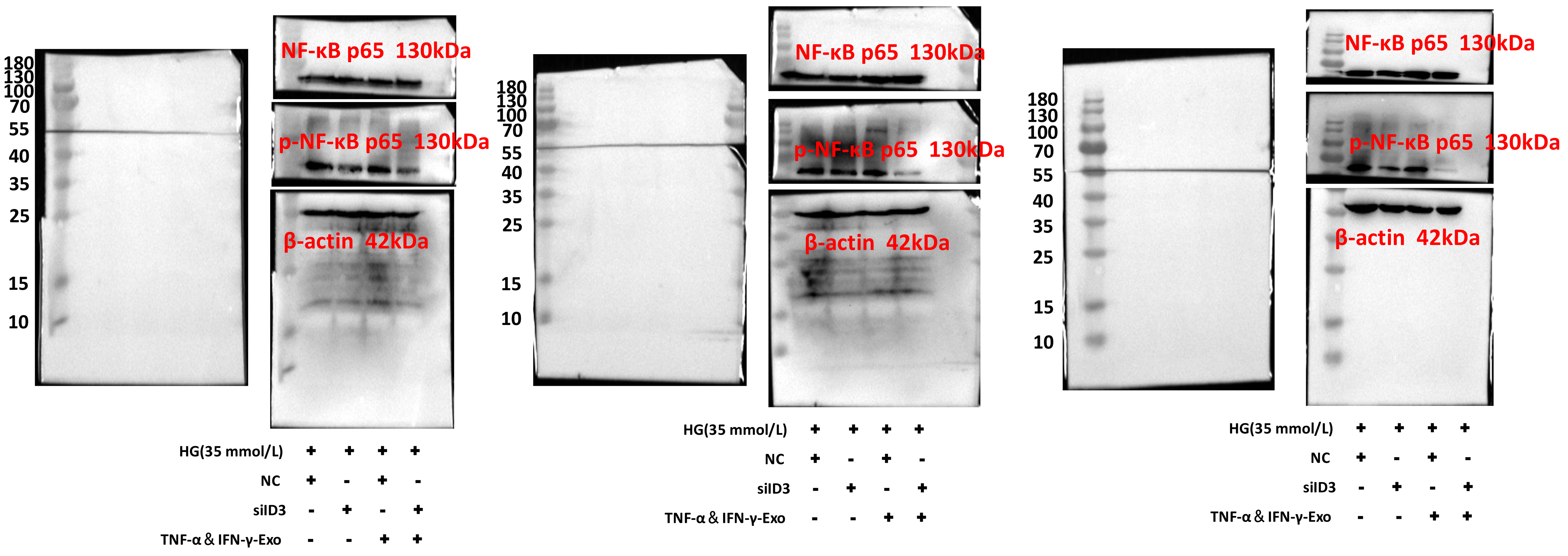

Supplementary Figure 17. After ID3 knockdown transfection of RAW 264.7 cells for 24h, followed by cultured with high glucose (35 mmol/L) for 48h, then treated with TNF-α&IFN-γ-Exo for 48h. The expression level of NF-κB p65 and p-NF-κB p65 was detected using WB.

Supplemental Table 1. Primary antibodies used in this study

Antibody name	dilution	isotype	manufacturer	product #	specificity
Rabbit Recombinant Monoclonal TSG101 antibody	1：1000	IgG	Abcam	ab125011	Mouse, Rat, Human
CD9 Rabbit mAb	1：1000	IgG	ABclonal	A19027	Human, Mouse
CD63 Rabbit mAb	1：1000	IgG	ABclonal	A19023	Human, Mouse, Rat
TNF-α Rabbit pAb	1：1000	IgG	ABclonal	A11534	Human, Mouse, Rat
IL1β Rabbit pAb	1：1000	IgG	ABclonal	A1112	Human, Mouse, Rat
IL10 Rabbit pAb	1：1000	IgG	ABclonal	A2171	Human, Mouse, Rat
IL6 Rabbit pAb	1：1000	IgG	ABclonal	A0286	Human, Mouse, Rat
Anti -beta Actin Rabbit pAb	1：1000	IgG	Service bio	GB11001-100	Human, Mouse, Rat
Anti-id3 mouse mAb	1：1000	IgG	SANTA CRUZ	sc-56712	Human, Mouse, Rat
Anti-Arginase 1 mouse mAb	1：1000	IgG	SANTA CRUZ	sc-47715	Human, Mouse, Rat
iNOS Rabbit mAb	1：1000	IgG	Cell Signaling Technology	13120	Mouse
Anti-Lamin A/C Antibody	1：1000	IgG	BOSTER	BA1227	Human, Mouse, Rat
HRP-conjugated Goat anti-Rabbit IgG (H+L)	1：2000	IgG	ABclonal	AS014	Rabbit
HRP-conjugated Goat anti-Mouse IgG (H+L)	1：2000	IgG	ABclonal	AS003	Mouse

Supplemental Table 2. Primers used for quantitative RT-PCR

Primers	Forward	Reverse
m-β-actin	GGCTGTATTCCCCTCCATCG	CCAGTTGGTAACAATGCCATGT
m-CD5L	TCATATCAGCCACCAGCATCAGAG	CACATTGAGCCAACGTGTCTTCC
m-Parp16	ATTCCCAGAAGCAGCCCAAGAG	TGAGCAGCAGCAGCAGATACAG
m-Neurog2	GGCGTCATCCTCCAACTCCAC	GCTGCCAGTAGTCCACGTCTG
m-ID3	CTGCCTGTCGGAACGTAGCC	AGTGGTTCATGTCGTCCAAGAGG
m-Dmpk	ATAAGTGGGACATGCTGAAGAG	CTCATCCTGGAAGGCAAAGT
m-Gbp7	TCCTGTGTGCCTAGTGGAAAA	CAAGCGGTTCATCAAGTAGGAT
h-β-actin	CATGTACGTTGCTATCCAGGC	CTCCTTAATGTCACGCACGAT
h-ID3	GAGAGGCACTCAGCTTAGCC	TCCTTTTGTCGTTGGAGATGAC
h-TNFα	AAGAATCCAAGGAAGTGGC	TCTATTAAGGTGGTGGCGA
h-IL-6	CCTGAACCTTCCAAAGATGGC	TTCACCAGGCAAGTCTCCTCA
h-CD206	TACCCCTGCTCCTGGTTTTT	CAGCGCTTGTGATCTTCATT
h-ARG1	GTGGAAACTTGCATGGACAAC	AATCCTGGCACATCGGGAATC

Supplemental Table 3. Information on ID3 knockdown targets

Species	Primer Name	Sequence
Homo sapiens	ID3-317-s	CGGAACUUGUCAUCUCCAA
	ID3-317-a	UUGGAGAUGACAAGUUCCG
Homo sapiens	ID3-175-s	CCGAGAGGCACUCAGCUUA
	ID3-175-a	UAAGCUGAGUGCCUCUCGG
Homo sapiens	ID3-213-s	GCGCGUCAUCGACUACAUU
	ID3-213-a	AAUGUAGUCGAUGACGCGC
Mus musculus	ID3-204-s	AAUCCUGCAGCGUGUCAUA
	ID3-204-a	UAUGACACGCUGCAGGAUU
Mus musculus	ID3-224-s	ACUACAUCCUCGACCUUCA
	ID3-224-a	UGAAGGUCGAGGAUGUAGU
Mus musculus	ID3-308-s	AGCUCACUCCGGAACUUGU
	ID3-308-a	ACAAGUUCCGGAGUGAGCU
